# Resting‐State EEG Microstates Parallel Age‐Related Differences in Allocentric Spatial Working Memory Performance

**DOI:** 10.1007/s10548-021-00835-3

**Published:** 2021-04-19

**Authors:** Adeline Jabès, Giuliana Klencklen, Paolo Ruggeri, Christoph M. Michel, Pamela Banta Lavenex, Pierre Lavenex

**Affiliations:** 1 Laboratory of Brain and Cognitive Development, Institute of Psychology, University of Lausanne, 1015 Lausanne, Switzerland; 2 Functional Brain Mapping Laboratory, University of Geneva, 1202 Geneva, Switzerland; 3 Faculty of Psychology, Swiss Distance University Institute, 3900 Brig, Switzerland

**Keywords:** Microstates, Electroencephalography, Spontaneous brain activity, Healthy aging, Working memory performance

## Abstract

Alterations of resting-state EEG microstates have been associated with various neurological disorders and behavioral states. Interestingly, age-related differences in EEG microstate organization have also been reported, and it has been suggested that resting-state EEG activity may predict cognitive capacities in healthy individuals across the lifespan. In this exploratory study, we performed a microstate analysis of resting-state brain activity and tested allocentric spatial working memory performance in healthy adult individuals: twenty 25–30-year-olds and twenty-five 64–75-year-olds. We found a lower spatial working memory performance in older adults, as well as age-related differences in the five EEG microstate maps A, B, C, C′ and D, but especially in microstate maps C and C′. These two maps have been linked to neuronal activity in the frontal and parietal brain regions which are associated with working memory and attention, cognitive functions that have been shown to be sensitive to aging. Older adults exhibited lower global explained variance and occurrence of maps C and C′. Moreover, although there was a higher probability to transition from any map towards maps C, C′ and D in young and older adults, this probability was lower in older adults. Finally, although age-related differences in resting-state EEG microstates paralleled differences in allocentric spatial working memory performance, we found no evidence that any individual or combination of resting-state EEG microstate parameter(s) could reliably predict individual spatial working memory performance. Whether the temporal dynamics of EEG microstates may be used to assess healthy cognitive aging from resting-state brain activity requires further investigation.

**Supplementary Information:**

The online version contains supplementary material available at 10.1007/s10548-021-00835-3.

## Introduction

Despite the on-going debate on the relevance of studying brain activity under resting-state conditions (Buckner and Vincent 2007; Campbell and Schacter 2017; Davis et al. 2017; Morcom and Fletcher 2007), several studies have shown important age-related differences in spontaneous EEG oscillations recorded when individuals are at rest (for a recent review see Anderson and Perone 2018). An alternative to the analysis of resting-state EEG in terms of frequency power modulation are EEG microstates, which represent short-lasting periods of synchronized activity of large-scale brain networks (Khanna et al. 2015; Koenig et al. 2005; Lehmann and Michel 2011; Michel and Koenig 2018). EEG microstates are characterized by a stable topography of the global scalp potential field within a millisecond resolution (Khanna et al. 2014). EEG microstates have been proposed to reflect the building blocks of information processing (Lehmann and Michel 2011; Michel and Koenig 2018). Accordingly, distinct microstate maps have been associated with visual imagery or abstract thinking (Lehmann et al. 1998), divergent beliefs (Schlegel et al. 2012), object/spatial visualization or verbalization (Milz et al. 2016a), perception of bodily signals (Pipinis et al. 2017), fluid intelligence (Santarnecchi et al. 2017; Zappasodi et al. 2019), and autobiographic memory retrieval or arithmetic processing (Brechet et al. 2019).

In addition, several studies have reported that resting-state EEG microstates are altered in neuropsychiatric and neurodegenerative disorders, such as Alzheimer’s disease (Musaeus et al. 2019; Smailovic et al. 2019), Parkinson’s disease (Chu et al. 2019), schizophrenia (Andreou et al. 2014; Baradits et al. 2020; da Cruz et al. 2020; de Bock et al. 2020; Giordano et al. 2018; Kindler et al. 2011; Koenig et al. 1999; Lehmann et al. 2005; Murphy et al. 2020a; Nishida et al. 2013; Soni et al. 2018; Strelets et al. 2003; Tomescu et al. 2014; Tomescu et al. 2015), multiple sclerosis (Gschwind et al. 2016), fibromyalgia (Gonzalez-Villar et al. 2020), panic disorder (Kikuchi et al. 2011), bipolar disorder (Damborska et al. 2019; Vellante et al. 2020), obsessive–compulsive disorder (Yoshimura et al. 2019), depressive disorder (Murphy et al. 2020b), narcolepsy (Drissi et al. 2016; Drissi et al. 2019), stroke (Zappasodi et al. 2017) or autism (D’Croz-Baron et al. 2019; Jia and Yu 2019; Portnova et al. 2020). EEG microstate analyses might therefore provide another tool for the development of a non-invasive assessment of healthy cognitive aging.

Healthy or normal cognitive aging is difficult to characterize and is essentially defined by exclusion, as the absence of evidence of mild cognitive impairment (MCI) or dementia, such as Alzheimer’s disease (Fabiani 2012; Jagust 2013; Rowe and Kahn 1987). Normal aging is associated with a variety of changes in cognitive capacities, one of which is an overall decline in working memory performance (Fabiani 2012; Jagust 2013). However, few studies have investigated age-related differences in EEG microstate maps across normal development and aging, and only one study has investigated the possible links between resting-state EEG microstates and non-pathological, age-related cognitive decline (Zanesco et al. 2020). There is to date no published study investigating the possible links between resting-state EEG microstates and spatial working memory performance in healthy individuals.

Koenig et al. (2002) analyzed the eyes-closed resting-state EEG of 496 participants (230 females) from 6 to 80 years of age. They computed three temporal parameters (mean duration, frequency and the percentage of time covered by each state) of the four canonical microstate maps (A, B, C, D), and showed that several microstate parameters followed distinct developmental trajectories. Although graphical data representations suggested distinct changes of the four maps from 21 to 80 years of age, the focus of the paper was on developmental stages, not aging, and no analyses comparing young and older adults were reported. Tomescu et al. (2018) analyzed the eyes-closed resting-state EEG of 179 participants (90 females) from 6 to 87 years of age. They computed three temporal parameters (mean duration, occurrence and map transitions) of the four canonical microstate maps (A, B, C, D), and reported age-related differences in the mean duration of map C. Specifically, they found a lower mean duration of map C in 61–87-year-olds, as compared to 31–60-year-olds. They also found lower transition probabilities from map C to map D, and from map D to map C in 61–87-year-olds, as compared to 31–60-year-olds. However, they did not compare young adults (20–30-year-olds) and older adults (61–87-year-olds). Zanesco et al. (2020) analyzed the eyes-open and eyes-closed resting-state EEG of 153 young participants (20–35 years of age; 45 females) and 74 older participants (59–77 years of age; 37 females). They computed three temporal parameters (GEV, mean duration, and occurrence) of five data-driven microstate maps (A, B, C, D, E) and assessed several cognitive, personality and mood traits using a standardized testing battery. They found age-related differences in GEV (in older participants: higher GEV for maps A and B, and lower GEV for maps C and E), mean duration (an overall increase with age) and occurrence (an overall decrease with age). They also found higher transition probabilities towards maps A and B and lower transition probabilities towards map C and from map C to map E in 59–77-year-olds, as compared to 20–35-year-olds. In addition, they showed that age and gender predicted more reliably the temporal dynamics of microstates than personality, mood or attentional performance.

Here, we aimed to explore potential differences in resting-state EEG microstates between healthy 20–30-year-old and 65–75-year-old adults and evaluate whether EEG microstates could be used to predict age-related differences in spatial working memory performance. To extend previous findings (Custo et al. 2017; Zanesco et al. 2020), we used a data-driven approach to provide a comprehensive description of the microstate maps that best explained resting-state brain dynamics. We measured allocentric spatial working memory performance, a capacity dependent on the integrity of the hippocampal formation (Banta Lavenex et al. 2014; Burgess 2006), which has been shown to decline with age (Klencklen et al. 2017a, b). We adapted standard EEG methods to record brain activity in mobile participants in a non-shielded environment, in both eyes closed and eyes open conditions. Consistent with previous studies, we found age-related differences in EEG microstates and spatial working memory performance. However, no individual or combination of resting-state EEG microstates parameter(s) could be used to predict individual spatial working memory performance in young or older adults.

## Materials and Methods

### Participants

Twenty young adults between 20 and 30 years of age (M 26.29, SD 3.57; 11 women) and 25 older adults between 65 and 75 years of age (M 71.75, SD 3.36; 8 women) were included in this study. Three other participants (one young and two older adults) were excluded due to the presence of too many residual artifacts in the EEG recordings. Participants were recruited via personal connections, social networks and flyers distributed through local senior organizations. Care was taken to recruit participants from all education levels. Exclusion criteria were subjective memory complaints and a history of learning disabilities, visual perception disabilities, left-handedness, birth complications, neurological medication, a history of neurological or psychiatric disease and trauma. All participants (except one young and one older adult) participated in previous studies (Klencklen et al. 2017a, b) and were screened at the time (2 years prior to the current experiment) for dementia by a neuropsychologist (G.K.), using a battery of tests: general cognitive status with the Mini Mental State Examination (MMSE; Folstein et al. 1975); the Progressive Matrice-12 (Raven et al. 2003); the Vocabulary, Digit Spans, Arithmetic and Similitude sub-tests from the Wechsler Adult Intelligence Scale-III (WAIS-III; Wechsler 1997); color vision with the Ishihara test (Ishihara 1917); and the Corsi Block-Tapping test Forward and Backward (Corsi 1972). For each test, older participants were found to be within 1.75 standard deviations of the norm for age-matched controls (Klencklen et al. 2017a, b). At the time of the present study, normal cognitive capacities were inferred using participants’ self-report, as well as by comparing the spatial working memory performance of older individuals who were tested in both the present study and the study 2 years prior (Klencklen et al. 2017a; paired t-tests: CBE: t_(22)_ = 1.664, p = 0.110; NET: t_(22)_ = 1.811, p = 0.084). We also compared the results of all the older participants tested in the current study with those of all the older participants tested previously (Klencklen et al. 2017a; unpaired t-tests. CBE: t_(57)_ = 0.508, p = 0.613; NET: t_(57)_ = 0.757, p = 0.452). Participants were tested for approximately 2 h between 8 a.m. and 8 p.m. All participants gave written informed consent prior to beginning the study and were compensated monetarily for their participation. Human subjects research was approved by the Cantonal Ethics Committee (Vaud, Switzerland, Protocol No. 384/15).

### Spatial Working Memory Task

**Fig. 1:**
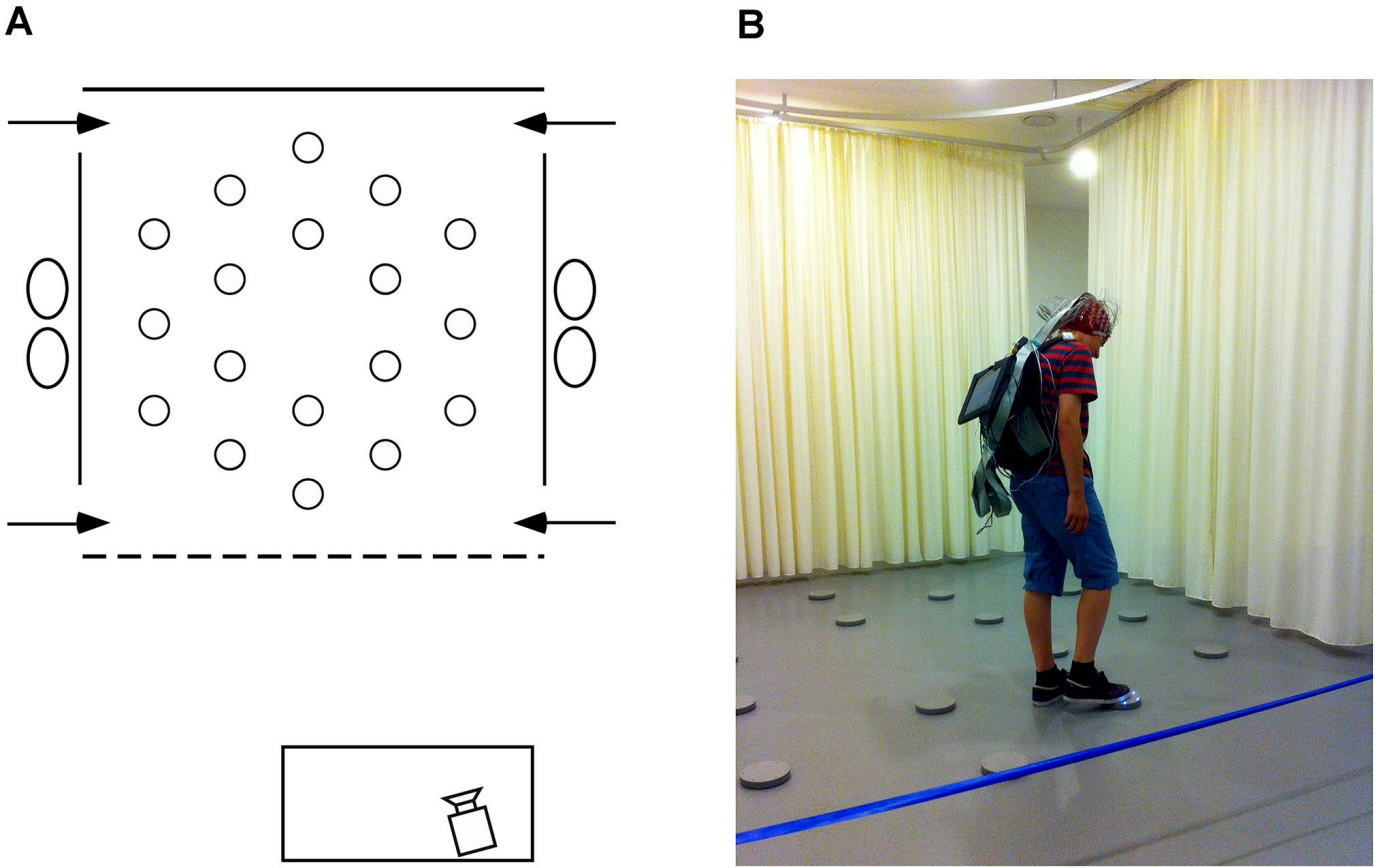
Testing environment. **a** Schematic, aerial view of the arena (3.64 × 3.64 m) within the experimental room (8 × 8 m). At each of the four near and far corners of the curtained arena was a 50 cm gap that served as one of the four different entry points (arrows) through which participants must pass in order to enter and exit the testing arena. Eighteen foot pads were regularly arranged on the floor of the arena. **b** Picture of the arena with a participant carrying the EEG backpack and touching an illuminating foot pad during the allocentric spatial working memory task

We measured allocentric spatial working memory performance, the ability to encode and recall on a trial-unique basis three locations defined with respect to their position relative to the environment, a capacity dependent on the integrity of the hippocampal formation (Banta Lavenex et al. 2014; Burgess 2006). Participants were tested at the University of Lausanne in a large square room (8 m × 8 m; Fig. 1a) containing many polarizing features such as a door, a table, chairs and folding room-dividing screens.

Detailed description of the testing facility and procedure has been published previously (Klencklen et al. 2017a, b). Briefly, within the room a 3.64 m × 3.64 m testing arena contained 18 visually identical, circular gray foot pads equipped with LED lights. Foot pads designated as goals by the experimenters would illuminate when touched lightly with the foot, but the light would extinguish as soon as the foot was removed from the pad. All testing was videotaped with a video camera located in front of the testing arena. Participants were given 10 trials during which they had to learn three predetermined goal locations on each trial. Each trial consisted of two phases: during the first encoding phase, participants had to explore the arena to discover the three goal locations, touching each disk in order to identify, learn and remember the locations of the illuminating disks. During a 90-s inter-phase interval, participants were required to mentally count backward by one from a predetermined number. This interference task was intended to suppress verbal rehearsal of the goal locations to be remembered, as working memory performance depends on the ability of individuals to resist interference to remember and use trial-unique information that must be distinguished from information acquired on previous trials (Banta Lavenex et al.^2014^; Bizon et al. 2012; Engle et al. 1999; Spellman et al. 2015). After the inter-phase interval, the recall phase began: participants were asked to indicate the three goal locations, in no particular order, by walking to each disk and stepping on it to illuminate it. In order to preclude participants from using an egocentric strategy to solve the task, they entered and exited the arena from different, pseudo randomly predetermined doors on every trial, as instructed by an experimenter during the task (Ribordy et al. 2013). Our task can thus test allocentric spatial learning and memory in a controlled, real-world laboratory environment in which participants have access to all sensory information, including vestibular and proprioceptive information, normally available when moving about in everyday life (in contrast to experiments carried out on a tabletop, a computer screen or in virtual environments).The same procedure was repeated for 10 trials with three new and non-adjacent goal locations predetermined pseudo-randomly for each trial.

### EEG Resting‐State Recordings and Analyses

#### Recordings

A 128-channel Biosemi Active Two system (Biosemi, Amsterdam, Netherlands) was used to record spontaneous resting-state brain activity just minutes before the spatial working memory task began. We used surgical caps designed following the BioSemi equiradial electrode placement system (‘ABC’ layout). Participants wore a backpack to carry the recording system, which allowed them to move freely and perform the spatial working memory task immediately after recording (Fig. 1b). A Sony tablet running the ActiView software (version 7.05) was fixed to the front side of the backpack, allowing constant monitoring of the EEG recording. For resting-state recording, participants were seated on a stool on the left side of the arena, where ambient electrical noise was minimal. Participants were instructed to relax and move as little as possible, fixing a cross made of tape on the back of a chair during the eye open condition. Six 1-min baseline recordings, alternating eyes-closed and eyes-open for 1 min each, were performed at a sampling rate of 2048 Hz before the spatial working memory task. The level of DC offset was checked (± 20 mV) before data collection. Right, left, and middle-orbital flat electrodes were used to monitor eye movements and blinks.

#### EEG Microstate Analyses

Signal pre-processing was performed with BrainVision Analyzer software 2.1 (Brain Products, München, Germany). A 1-Hz high-pass filter (IIR Filter Butterworth, order 2) and a 50-Hz Notch filter were applied. Data were then visually inspected and segments containing residual artifacts (except blinks and eye movement artifacts) were manually removed. Independent Component Analysis (ICA) was performed to correct eye movements, blinks and cardiac artifacts. Topographic interpolation (3D spherical spline) was used to correct channels that were noisy throughout the recording (max. 10). Then, a 40-Hz low pass filter (IIR Filter Butterworth, order 2) was applied. Data were average-referenced and downsampled to 128 Hz. Eyes closed and eyes open preprocessed data were then segmented and exported for further analysis.

**Fig. 2:**
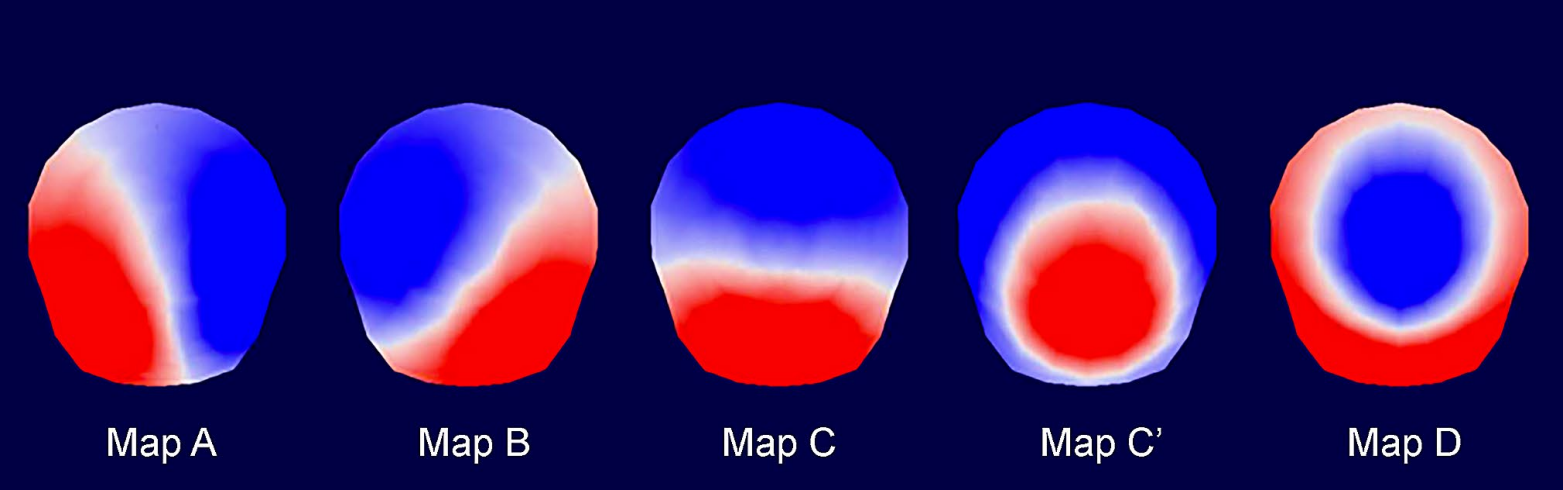
Five dominant cluster maps from all participants in the eyes closed condition optimally described the data (maps A, B, C, C′, and D, following the description of Michel and Koenig 2018)

Microstate analyses were performed using the freely available software Cartool, version 3.60 (https://sites.google.com/site/cartoolcommunity/ Brunet et al. 2011). The Global Field Power (GFP), i.e., the spatial standard deviation of EEG signal across all electrodes, is associated with a stable EEG topography around its peak and therefore shows an optimal signal-to-noise ratio in the EEG (Khanna et al. 2015; Koenig et al. 2005; Michel and Koenig 2018; Michel et al. 2009). Therefore, the EEG signal was extracted at the GFP peaks and submitted to a modified k-means cluster analysis (Brunet et al. 2011; Pascual-Marqui et al. 1995). This procedure allows the identification of the microstate map topographies that best contribute to the global explained variance. The cluster analysis was first applied at the individual level and then at the group level. In a first step, using a data-driven approach, we computed the dominant cluster maps using optimal clustering criterion for the young and older groups, and for the eyes closed and eyes open conditions, separately. We identified five maps that best explained our data and compared them across age groups and eye conditions using a topographic ANOVA (Koenig et al. 2014). We did not find consistent differences between eye conditions or between groups. Clustering solutions and statistics for each condition and group are provided in Supplementary Material 1. In a second step, and for further analysis as these maps did not differ between eye conditions or between groups, we computed the dominant cluster maps using optimal clustering criterion for all participants combined in the eyes closed condition (Fig. 2). This was a conservative choice simplifying comparisons with previous findings reported in the literature, which have been most often obtained in the eyes closed condition. The five microstate maps identified (A, B, C, C′, D) explained 78% of the global variance of the individual data, before the backfitting process performed next. Finally, every time point of the individual data was assigned to the one map of the five microstate maps with which it correlated best (backfitting process) (Brunet et al. 2011).

Following this backfitting process, we extracted three parameters for each microstate map: (1) the global explained variance (GEV), which is the sum of the explained variance of a given map weighted by the global field power. GEV represents how well a map explains the data both in terms of strength and in terms of frequency of occurrence; (2) the mean duration (mDur), which is the averaged amount of time in milliseconds that a given map was present without interruption; and (3) the occurrence (Occ), which is the number of times a given microstate occurred per second. The transition probabilities between each of the five maps were also analyzed using a Markov chain approach (Lehmann et al. 2005). For each subject and transition pair (for example AB), we computed the number of transitions from A towards B and normalized it by the number of transitions towards all possible maps from A (e.g., towards B, C, C′, and D). The transition probabilities were calculated for 20 pairs of maps (AB, AC, AC′, AD, BA, BC, BC′, BD, etc.). In order to establish that the observed differences in microstate map transitions between eyes-open and eyes-closed conditions in young and older individuals cannot be reduced to differences in map occurrences, a randomized procedure was used, as described in (Lehmann et al. 2005; Nishida et al. 2013). For each group (young and older) and condition (eyes open and eyes closed), observed and expected (i.e., based on the occurrence of the microstate maps) transitions were averaged across subjects and their difference evaluated with the Chi square distance. This distance was statistically compared to a null model distribution of Chi square distances obtained by random permutation (5000 times) of the observed and expected transitions at the subject level. A significant result (*p* < 0.05) indicates that the transition from one microstate map to another does not occur randomly, and therefore is not simply based on microstate map occurrences.

### Statistical Analyses

General Linear Model analyses with repeated-measures, t-tests and Pearson’s correlations were performed using IBM SPSS Statistics for Macintosh, version 25.0 (IBM Corp., Armonk, NY). Principal component analyses and multiple regression analyses were performed with the R software, version 3.3.3 (R Core Team, 2016). Descriptive statistics are provided as Supplementary Material 2.

#### Microstates

For each microstate parameter (GEV, mDur, Occ), we performed a GLM analysis (ANOVA) with age as a between-subjects factor, and five microstate maps (A, B, C, C′, D) and two eye conditions (closed, open) as repeated measures. We also performed a GLM analysis with age as a between-subjects factor, and microstate transition probabilities (20 pairs) and two eye conditions (closed, open) as repeated measures. Independent samples t-tests were used as post hoc analyses to compare map variables and transition probabilities between young and older adults in each eye condition. Paired samples t-tests were used to compare maps parameters between eye conditions, within age groups. As we found only marginal effects of sex on microstate parameters [sex effect for mDur: F_(1,41)_ = 7.361, p = 0.010, η^2^_p_ = 0.152; interaction between map × eye condition × sex for Occ: F_(3.270,134.057)_ = 3.280, p = 0.020, η^2^_p_ = 0.074], and no effect of sex on transition probabilities, data from men and women were combined. Statistical significance level was set at *p* < 0.05 for all analyses. For ANOVAs, we report effect size with η^2^_p_ [partial eta squared: ${SS}_{effect}/({SS}_{effect} + {SS}_{total}$), as reported by SPSS 25.0]. We report effect size with Cohen’s d_s_ (${d}_{s}$ = $t * \sqrt{\frac{1}{{n}_{1}} + \frac{1}{{n}_{2}}}$) for independent samples t-tests, and Cohen’s d_z_ for paired samples t-tests (${d}_{z}$ = $t / \sqrt{n}$) (Lakens 2013).

#### Spatial Working Memory

The following two measures were used to characterize spatial working memory performance: (1) the number of goal disks visited before making an error (CBE: correct before error), an estimate of memory capacity; and (2) the number of errorless trials (NET), an estimate of perfect memory (Banta Lavenex et al. 2014). Independent samples t-tests were used to compare these measures between young and older adults. Statistical significance level was set at *p* < 0.05 and effect size was reported with Cohen’s d_s_, as described above.

#### Microstates and Spatial Working Memory

In order to test whether different resting-state microstate signatures may predict allocentric spatial working memory performance in young and older adults, we performed a Principal Component Analysis (PCA, no rotation; Abdi and Williams 2010) on all 70 microstate parameters (30 variables and 40 transitions) for all participants. The same analysis was performed excluding the 40 microstate transition variables. As both analyses provided similar results and did not change any conclusions, we reported only the former. Results of Horn’s Parallel Analysis for component retention (2100 iterations, 95 centile estimate) revealed five components with an adjusted Eigenvalue > 1. These five components explained 77.37% of the total variance and were retained for further analysis. We then performed multiple regression analyses with the five microstate components extracted from the PCA and the two measures of spatial working memory performance (CBE and NET). Finally, these five-component models were simplified using a step by step minimization of Akaike Information Criterion (AIC, Sakamoto et al. 1986). These parsimonious models described the data as well as the five-component models [CBE: ΔR^2^ = 0.03, F_(9, 33)_ = 0.354, p = 0.948; NET: ΔR^2^ = 0.05, F_(9, 33)_ = 0.456, p = 0.893]. In addition, we performed two Bayesian multiple regressions analyses with the five microstate components extracted from the PCA and the two measures of spatial working memory performance (CBE and NET).

## Results

### Resting‐State EEG Activity

The five microstate maps from all participants in the eyes closed condition are shown in Fig. 2. These five maps are largely similar to the one described previously (Custo et al. 2017; Michel and Koenig 2018; Zanesco et al. 2020) and labelled as maps A, B, C, C′ and D, following the nomenclature defined by Michel and Koenig (2018). In a recent review, these authors highlighted that in studies using the four canonical maps (A, B, C, D) microstate C is in fact a combination of two distinct maps as revealed by a combined EEG-fMRI study (Custo et al. 2017). Note that map C′ in the current study seems to correspond to microstate 3 of Britz et al. (2010), map F of Custo et al. (2017) and map E of Zanesco et al. (2020).

#### Global Explained Variance: GEV

**Fig. 3:**
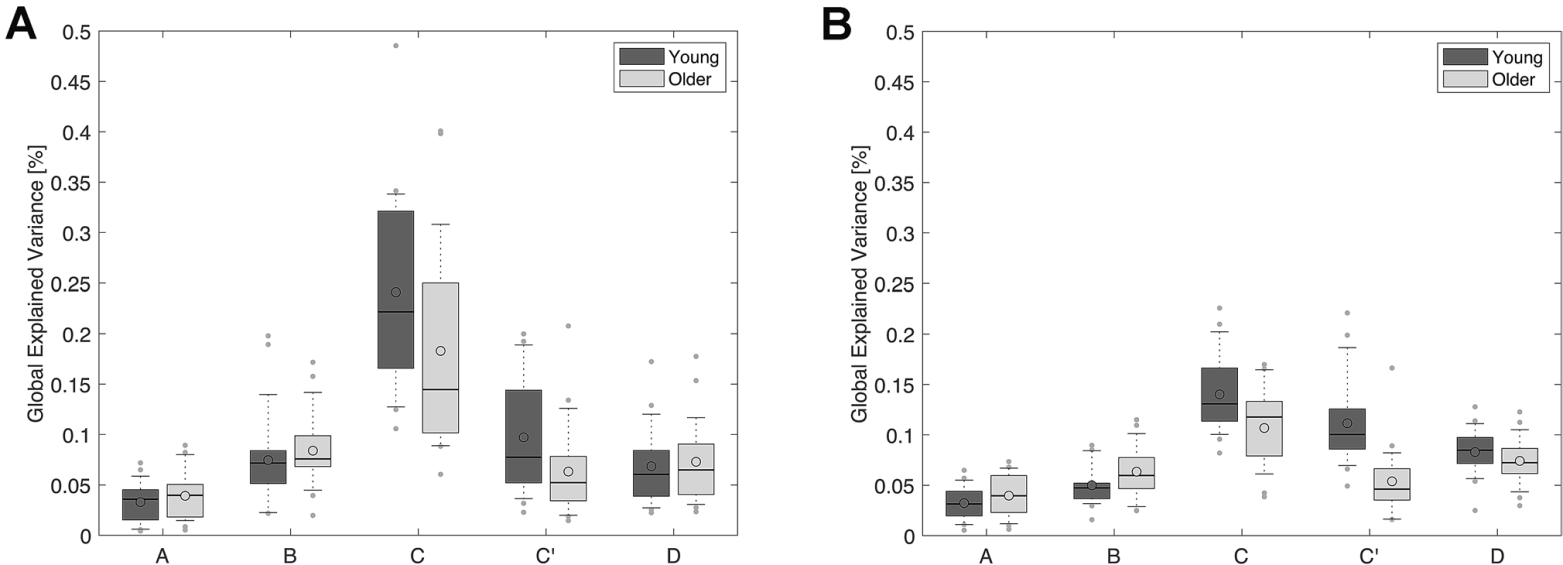
Global explained variance. GEV, measured across the scalp, was overall lower for maps C and C′ in 65–75-year-old adults (Older) than in 20–30-year-old adults (Young), in the eyes closed (**a**) and eyes open (**b**) conditions. Dark grey plots: young adults; light grey plots: older adults. Lower and upper box boundaries represent the 25th and 75th percentiles, respectively; the line and the empty circle inside the box represents the median and the mean, respectively; the lower and upper error lines represent the 10th and the 90th percentiles, respectively; finally, the filled gray circles are data points falling outside the 10th and 90th percentiles

There was a main effect of microstate maps for GEV [Fig. 3; F_(2.619, 112.624)_ = 70.604, p < 0.001, Greenhouse-Geisser correction, η^2^_p_ = 0.621] and an interaction between maps and age groups [F_(2.619, 112.624)_ = 5.912, p = 0.001, η^2^_p_ = 0.121]. Interestingly, we also found an overall lower GEV in older adults, as compared to young adults, across the five maps and the two eye conditions [age groups: F_(1,43)_ = 17.966, p < 0.001, η^2^_p_ = 0.295], and no interaction between eye conditions and age groups [F_(1, 43)_ = 0.096, p = 0.758, η^2^_p_ = 0.002].

In the eyes closed condition, map C best explained the data for both age groups. In contrast, in the eyes open condition, whereas map C best explained the data for older adults, maps C and C′ best explained the data for young adults (Supplementary Material 3). The GEV of map A was the lowest for both age groups. The GEV of map C was lower for older adults than for young adults [t_(43)_ = -2.975, p = 0.005, d_s_ =0.893] in the eyes open condition, whereas the difference was marginal in the eyes closed condition [t_(43)_ = − 1.956, p = 0.057, d_z_ = 0.587]. The GEV of map C′ was lower for older adults than for young adults, in both the eyes open condition [t_(43)_ = − 5.175 p < 0.001, d_z_ = 1.553] and the eyes closed condition [t_(35.466)_ = − 2.192, p = 0.035, d_s_ = 0.658]. In sum, microstates C and C′, which have been linked to neuronal activity in frontal and parietal brain regions (Custo et al. 2017), appeared to contribute less to GEV in older adults than in young adults. In other words, microstates C and C′ contributed less to overall resting-state brain dynamics in older adults.

We also found an overall lower GEV (including maps A, B, C, C′, and D) in the eyes open condition, as compared to the eyes closed condition, for both young and older adults [Fig. 3; F_(1, 43)_ = 78.763, p < 0.001, η^2^_p_ = 0.647; eye conditions × age groups: F_(1, 43)_ = 0.096, p = 0.758, η^2^_p_ = 0.002]. We found an interaction between eye conditions and maps [F_(1.781, 76.581)_ = 23.506, p < 0.001, η^2^_p_ = 0.353], but no interaction between maps, eye conditions, and age groups: F_(1.781, 76.581)_ = 1.244, p = 0.291, η^2^_p_ = 0.028. The GEV of map B was lower in the eyes open condition than in the eyes closed condition for both young adults [t_(19)_ = 2.814, p = 0.011, d_z_ = 0.629] and older adults [t_(24)_ = 3.245, p = 0.003, d_z_ = 0.649]. In addition, the GEV of map C was lower in both young and older adults in the eyes open condition, as compared to the eyes closed condition [young: t_(19)_ = 5.070, p < 0.001, d_z_ = 1.134; older: t_(24)_ = 3.827, p = 0.001, d_z_ = 0.675].

#### Mean Duration: mDur

**Fig. 4:**
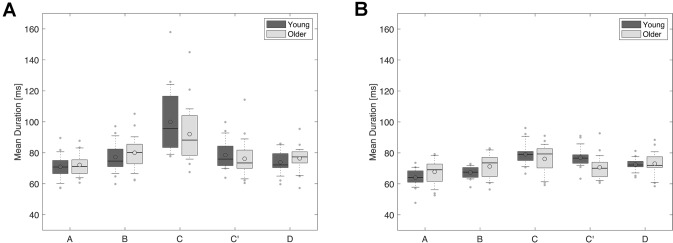
Mean duration. mDur of maps, measured across the scalp, was similar in 65–75-year-old adults (Older), as compared to 20–30-year-old adults (Young), in the eyes closed (**a**) and eyes open (**b**) conditions. Dark grey plots: young adults; light grey plots: older adults. Lower and upper box boundaries represent the 25th and 75th percentiles, respectively; the line and the empty circle inside the box represents the median and the mean, respectively; the lower and upper error lines represent the 10th and the 90th percentiles, respectively; finally, the filled gray circles are data points falling outside the 10th and 90th percentiles

There was a main effect of maps for mDur [Fig. 4; F_(2.647, 113.826)_ = 43.673, p < 0.001, η^2^_p_ = 0.504] and an interaction between maps and age groups [F_(2.647, 113.826)_ = 4.121, p = 0.011, η^2^_p_ = 0.087]. In the eyes closed condition, map C exhibited the longest mDur for both age groups. In contrast, in the eyes open condition, map C exhibited the longest mDur for older adults, whereas maps C and C′ exhibited the longest mDur for young adults (Supplementary Material 3). The mDur of map A was the shortest for both age groups. There were no age group differences in overall mDur in either eye condition [F_(1,43)_ = 0.106, p = 0.747, η^2^_p_ = 0.002; eye conditions × age groups: F_(1,43)_ = 0.063, p = 0.803, η^2^_p_ = 0.001], revealing that the average amount of time maps were present without interruption during resting-state was similar in 65–75-year-old adults, as compared to 20–30-year-old adults.

The mDur of microstate maps was shorter in the eyes open condition than in the eyes closed condition [F_(1,43)_ = 56.143, p < 0.001, η^2^_p_ = 0.566], for both young and older adults [eye conditions × age groups: F_(1,43)_ = 0.063, p = 0.803, η^2^_p_ = 0.001]. However, there was an interaction between eye conditions and maps [F_(1.978, 85.040)_ = 17.625, p < 0.001, η^2^_p_ = 0.291], but no interaction between maps, eye conditions and age groups [F_(1.978, 85.040)_ = 1.294, p = 0.279, η^2^_p_ = 0.029]. The mDur of map A was shorter in the eyes open condition as compared to the eyes closed condition, in both young adults [t_(19)_ = − 3.925, p = 0.001, d_z_ = 0.878] and older adults [t_(24)_ = − 3.909, p = 0.001, d_z_ = 0.782]. The mDur of map B was also shorter in the eyes open condition as compared to the eyes closed condition, in both young adults [t_(19)_ = − 4.757, p < 0.001, d_z_ = 1.064] and older adults [t_(24)_ = − 5.216, p < 0.001, d_s_ = 1.043]. The mDur of map C was shorter in the eyes open condition as compared to the eyes closed condition, in both young adults [t_(19)_ = − 4.892, p < 0.001, d_z_ = 1.094] and older adults [t_(24)_ = − 4.112, p < 0.001, d_z_ = 0.822]. In contrast, the mDur of map C′ was shorter in the eyes open condition than in the eyes closed condition for older adults [t_(24)_ = − 2.147, p = 0.042, d_z_ = 0.429], but not for young adults [t_(19)_ = − 0.812, p = 0.427, d_z_ = 0.182].

#### Occurrence: Occ

**Fig. 5:**
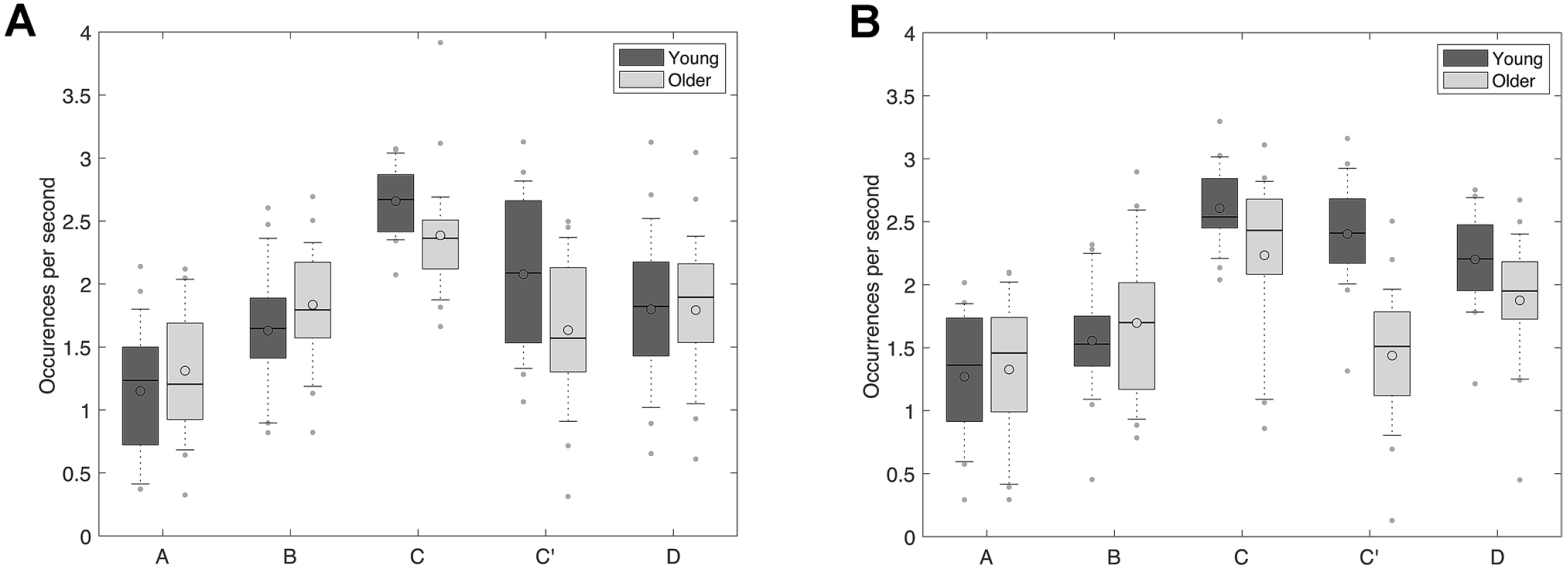
Occurrence. Occ of maps C and C′ was lower in 65–75-year-old adults (Older) than in 20–30-year-old adults (Young), in the eyes closed (**a**) and eyes open (**b**) conditions. Occ of map D was lower in older adults in the eyes open condition. Dark grey plots: young adults; light grey plots: older adults. Lower and upper box boundaries represent the 25th and 75th percentiles, respectively; the line and the empty circle inside the box represents the median and the mean, respectively; the lower and upper error lines represent the 10th and the 90th percentiles, respectively; finally, the filled gray circles are data points falling outside the 10th and 90th percentiles

Occ differed between maps [Fig. 5; F_(3.254, 139.940)_ = 51.020, p < 0.001, η^2^_p_ = 0.543] and there was an interaction between maps and age groups [F_(3.254, 139.940)_ = 8.407, p < 0.001, η^2^_p_ = 0.164]. In both young and older adults map C exhibited the highest Occ, whereas map A exhibited the lowest Occ. Interestingly, older adults exhibited an overall lower map Occ than young adults [F_(1, 43)_ = 5.511, p = 0.024, η^2^_p_ = 0.114], which was influenced by eye condition [eye conditions × age groups: F_(1, 43)_ = 6.921, p = 0.012, η^2^_p_ = 0.139].

Occ of map C was lower in older adults than in young adults, in the eyes open condition [t_(36.602)_ = − 2.579, p = 0.014, d_s_ = 0.774], and in the eyes closed condition [t_(43)_ = − 2.356, p = 0.023, d_s_ = 0.707]. Similarly, Occ of map C′ was lower in older adults than in young adults in the eyes open condition [t_(43)_ = − 6.841, p < 0.001, d_s_ = 2.052], and in the eyes closed condition [t_(43)_ = − 2.563, p = 0.014, d_s_ = 0.769]. Finally, Occ of map D was lower in older adults than in young adults in the eyes open condition [t_(43)_ = − 2.529, p = 0.015, d_s_ = 0.759], but not in the eyes closed condition [t_(43)_ = − 0.035, p = 0.972, d_s_ = 0.011]. In sum, Occ of maps C, C′ and D, which have been linked to neuronal activity in the frontal and parietal brain regions (Custo et al. 2017), was lower in 65–75-year-old adults than in 20–30-year-old adults.

There was no main effect of eye conditions for Occ [F_(1, 43)_ = 0.616, p = 0.437, η^2^_p_ = 0.014]. There was, however, an interaction between eye conditions and maps for both age groups [F_(3.416, 146.883)_ = 5.522, p = 0.001, η^2^_p_ = 0.114; maps × eye conditions × age groups: F_(3.416, 146.883)_ = 2.543, p = 0.051, η^2^_p_ = 0.056]. Whereas Occ of map B was marginally lower in the eyes open condition than in the eyes closed condition [t_(44)_ = − 2.024, p = 0.049, d_z_ = 0.302], Occ of map D was higher in the eyes open condition than in the eyes closed condition [t_(44)_ = 2.721, p = 0.009, d_z_ = 0.508].

### Microstates Transitions Probabilities

**Fig. 6:**
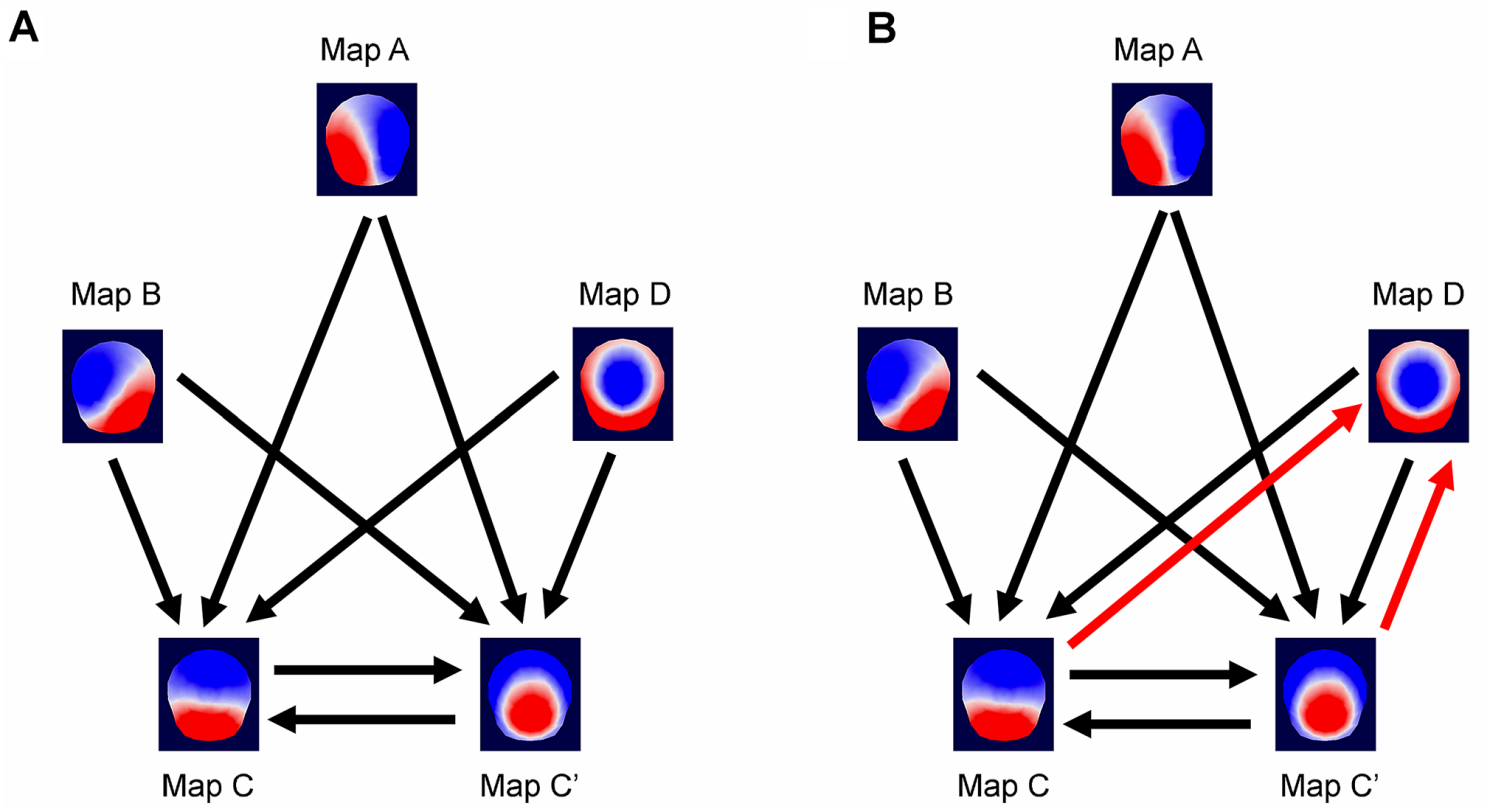
The probability to transition toward maps C and C′ was lower in 65–75-year-old adults (Older) than in 20–30-year-old adults (Young), in the eyes closed condition (**a**). The probability to transition toward maps C, C′ and D was lower in 65–75-year-old adults (Older) than in 20–30-year-old adults (Young), in the eyes open conditions (**b**). Black and red arrows: statistically significant decrease in older adults (p < 0.05; numerical values are provided in the main text). Red arrows highlight differences between eyes closed and eyes open conditions (Color figure online)

There were differences in the probability of transitions between different microstate maps [Fig. 6; Supplementary Material 3; F_(4.434, 190.681)_ = 47.704, p < 0.001, η^2^_p_ = 0.526], with overall higher transition probabilities toward maps C, C′ and D. There was also a main effect of age groups [F_(1, 43)_ = 9.478, p = 0.004, η^2^_p_ = 0.181], which was consistent across eye conditions [eye conditions × age groups: F_(1, 43)_ = 1.748, p = 0.193, η^2^_p_ = 0.039]. However, there was an interaction between transitions and age groups [F_(4.434, 190.681)_ = 7.068, p < 0.001, η^2^_p_ = 0.141].

In the eyes closed condition (Fig. 6a), the probability to transition from any map toward maps C and C′ was overall lower in older adults than in young adults. In the eyes open condition (Fig. 6b), the probability to transition from any map toward maps C, C′ and D was overall lower in older adults than in young adults. Altogether, these analyses revealed that the network dynamics differed between age groups. There were lower transition probabilities towards maps C, C′ and D, which have been linked to neuronal activity in frontal and parietal brain regions (Custo et al. 2017), in 65–75-year-old adults than in 20–30-year-old adults.

There were lower transition probabilities in the eyes open condition than in the eyes closed condition [F_(1, 43)_ = 70.478, p < 0.001, η^2^_p_ = 0.621], for both young and older adults [eye conditions × age groups: F_(1, 43)_ = 1.748, p = 0.193, η^2^_p_ = 0.039]. There was an interaction between eye conditions and transitions [F_(4.145, 178.246)_ = 8.629, p < 0.001, η^2^_p_ = 0.167], but no interaction between transitions × eye conditions **×** age groups [F_(4.145, 178.246)_ = 0.723, p = 0.582, η^2^_p_ = 0.017]. There were lower transition probabilities toward maps A, B, C and C′ in the eyes open condition than in the eyes closed condition. In addition, the transition probabilities from maps B and C toward map D were lower in the eyes open condition than in the eyes closed condition. In contrast, the transitions probabilities from maps A and C′ toward map D did not differ between eye conditions.

Randomization tests comparing the observed transitions probabilities and the expected probabilities based on the occurrence of the five maps showed that the observed transition probabilities were not significantly different from the expected ones in the eyes open and eyes closed conditions for the older and younger individuals (p > 0.3). Thus, it is possible that the age-related differences in observed transitions reported here may simply be explained by differences in the occurrence of the maps.

### Allocentric Spatial Working Memory Performance

**Fig. 7:**
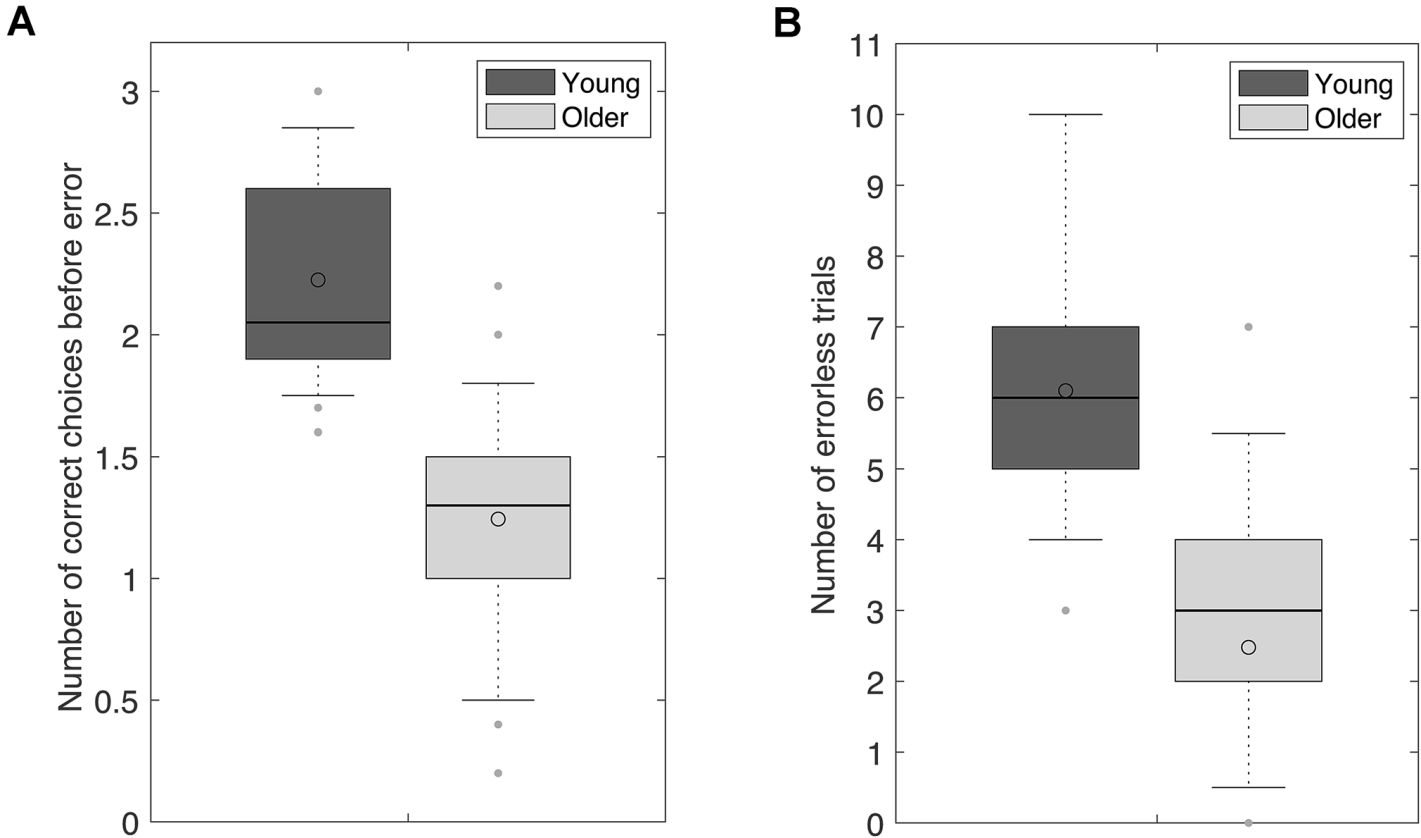
Allocentric spatial working memory performance was lower in 65–75-year-old adults (Older) than in 20–30-year-old adults (Young). **a** Number of correct choices before erring (CBE). **b** Number of errorless trials (NET). Dark grey plots: young adults; light grey plots: older adults. Lower and upper box boundaries represent the 25th and 75th percentiles, respectively; the line and the empty circle inside the box represents the median and the mean, respectively; the lower and upper error lines represent the 10th and the 90th percentiles, respectively

The two behavioral measures revealed a lower spatial working memory performance in older adults. Older adults made fewer correct choices before erring than young adults [Fig. 7a; CBE: t_(43)_ = − 7.180, p < 0.001; d_s_ = 2.154]. Similarly, older adults completed fewer errorless trials than young adults [Fig. 7b; NET: t_(43)_ = − 6.141, p < 0.001; d_s_ = 1.842].

### Principal Component and Multiple Regression Analyses

In order to determine whether some resting-state microstates signatures might predict allocentric spatial working memory performance in young or older adults, we performed a Principal Component Analysis (PCA) including the 70 microstates parameters reported above (30 microstates variables and 40 microstates transitions variables). Horn’s Parallel Analysis revealed five components, which explained 77.37% of the total variance. The variables that contributed to each of the five components retained in the PCA analysis are listed in Table 1.

**Table 1:** Microstate parameters that contributed to the five components retained from the Principal Component Analysis (PCA)

Eyes closed condition									Eyes open condition							
	Variables			Transition toward					Variables			Transition toward				
Comp. 1																
Map A	GEV	mDur	Occ	–					GEV	mDur	Occ	–	B	C	Cp	D
Map B				A	–					mDur	Occ	A	–	C	Cp	D
Map C				A		–				mDur	Occ	A	B	–	Cp	D
Map Cp				A			–			mDur	Occ	A	B	C	–	D
Map D				A				–		mDur		A	B	C	Cp	–
Comp. 2																
Map A				–	B		Cp		GEV			–			Cp	
Map B	GEV		Occ		–		Cp		GEV		Occ		–		Cp	
Map C						–	Cp							–	Cp	D
Map Cp	GEV		Occ			C	–		GEV	mDur	Occ				–	D
Map D							Cp	–			Occ				Cp	–
Comp. 3																
Map A				–		C						–				
Map B					–	C							–			
Map C	GEV	mDur			B	–								–		
Map Cp						C	–	D							–	
Map D	GEV	mDur	Occ			C		–								–
Comp. 4																
Map A				–			Cp					–				
Map B					–		Cp	D					–			
Map C						–	Cp	D						–		
Map Cp	GEV	mDur	Occ				–								–	
Map D	GEV						Cp	–	GEV							–
Comp. 5																
Map B		mDur			–								–			
Map Cp					B		–								–	
Map D					B			–								–

*GEV* global explained variance is the sum of the explained variance of a given map weighted by the global field power, *mDur* mean duration is the averaged amount of time in milliseconds that a given map was present without interruption, *Occ* occurrence is the number of times a given microstate occurred per second

Multiple regression analyses revealed that none of the PCA components accounted for spatial working memory performance (Table 2), as assessed by the number of correct choices before erring (CBE) or the number of errorless trials (NET). Moreover, there was no interaction between PCA components and age groups, for either measure of spatial working memory performance. No interactions between EEG components and age groups were retained in the simplified models. Bayesian multiple regression analyses equally showed the absence of association between the spatial working memory task parameters and the microstates predictors (Supplementary Material 4).

**Table 2:** Multiple regression analyses investigating the possible links between the microstate components extracted using PCA and allocentric spatial working memory performance (CBE, NET)

	CBE			NET		
	β	t	p	β	t	p
5-Component model						
Age group	− 1.54	− 4.994	**< 0.001**	− 1.48	− 4.548	**< 0.001**
Comp. 1	0.11	0.799	0.430	0.18	1.292	0.205
Comp. 2	− 0.11	− 0.726	0.473	− 0.17	− 1.075	0.290
Comp. 3	− 0.05	− 0.453	0.653	− 0.06	− 0.502	0.619
Comp. 4	− 0.14	− 1.220	0.231	− 0.12	− 0.946	0.351
Comp. 5	− 0.08	− 0.673	0.505	− 0.05	− 0.425	0.674
Age × Comp.1	− 0.18	− 0.683	0.500	− 0.09	− 0.319	0.752
Age × Comp.2	0.21	0.703	0.487	0.18	0.563	0.577
Age × Comp.3	0.01	0.028	0.978	− 0.03	− 0.118	0.907
Age × Comp.4	− 0.02	− 0.085	0.933	0.20	0.809	0.424
Age × Comp.5	− 0.02	− 0.080	0.937	0.02	0.100	0.921
F_(11, 33)_		4.59	**< 0.001**		3.83	**0.001**
Adj. R^2^		0.47			0.41	
R^2^		0.60			0.56	
Simplified model						
Age group	− 1.44	− 7.113	**< 0.001**	− 1.27	− 5.764	**< 0.001**
Comp. 1	–	–	–	0.20	1.826	0.075
Comp. 4	− 0.15	− 1.437	0.158	–	–	–
F_(2, 42)_		27.45	**< 0.001**		21.54	**< 0.001**
Adj. R^2^		0.55			0.48	
R^2^		0.57			0.51	

*P* values in bold are considered statistically significant

In sum, the present data did not reveal resting-state microstate variables that could be linked and therefore predict spatial working memory performance in healthy young or older adults.

## Discussion

### Age‐Related Differences in Resting‐State Brain Activity

Consistent with previous studies (Koenig et al. 2002; Tomescu et al. 2018; Zanesco et al. 2020), we found age-related differences in resting-state brain microstate dynamics. Moreover, we found age-related differences in resting-state microstates, which were dependent on the conditions of EEG recordings (eyes open versus eyes closed). Specifically, in the eyes closed condition, we found a lower occurrence of map C, and a lower GEV and occurrence of map C′ in older adults than in young adults. In the eyes open condition, we found a lower GEV and occurrence of maps C and C′, and a lower occurrence of map D in older adults than in young adults. In addition, while there was a higher probability to transition from any map towards maps C, C′ and D across age groups, the probability to transition toward maps C and C′ (and map D in the eyes open condition) was lower in older adults than in young adults. Thus, the most consistent age-related differences were observed for maps C and C′, two microstates which have been linked to neuronal activity in the frontal and parietal brain regions (Custo et al. 2017). However, none of the microstate parameters measured in the current study were correlated with, and could therefore be used to predict, allocentric spatial working memory performance in either young or older healthy adults.

#### Eyes Open Versus Eyes Closed

To our knowledge, only two prior studies described microstate differences between eyes closed and eyes open conditions at rest. These studies provided somewhat contrasting results. In the current study, we reported lower GEV (driven by maps B and C) and mDur (driven by maps A, B, C and C′) in the eyes open condition as compared to the eyes closed condition, and no main effect of eye condition for Occ (but marginally lower Occ for map B and higher Occ for map D).

Seitzman et al. (2017), considering four canonical microstates (A, B, C, D) in adults from 18 to 35 years of age also found overall lower GEV and mDur in the eyes open condition as compared to the eyes closed condition, but found an overall higher Occ in the eyes open condition (particularly for map B). In contrast, Zanesco et al. (2020), considering five data-driven microstates (A, B, C, D, E) in 20–35- and 59–77-year-old adults, mainly reported lower GEV, mDur and Occ of map C in the eyes open condition as compared to the eyes closed condition, and higher GEV, mDur, and Occ of map E (corresponding to our map C′) in the eyes open condition as compared to the eyes closed condition. In addition, we found lower transition probabilities in the eyes open condition, whereas Seitzman et al. (2017) did not report differences in microstate transitions between eyes open and eyes closed conditions. Zanesco et al. (2020) found a lower transition probability towards maps C and D in the eyes open conditions, and a higher transition probability toward map E (corresponding to our map C′) in the eyes open condition.

As previously discussed by Seitzman et al. (2017), such differences between experimental studies may depend on the activation of the visual system. Thus, studies using slightly different instructions in any particular eye condition may not be directly comparable. For example, whereas we instructed our participants to sit quietly during recording, and to fix a cross taped on the back of a chair in a lit room during the eyes open condition, Seitzman et al. (2017) asked their participants to let their mind wander and fix a cross on a computer screen, and Zanesco et al. (2020) asked participants to sit still and fix a black cross on a white background on a computer screen. Similarly, it is well established that EEG activity will depend on the state of wakefulness (Cantero et al. 2002). Whereas we collected 6 × 1 min EEG recordings in alternating eyes open and eyes closed conditions, Seitzman et al. (2017) used three separate 2-min trials for each eye condition with a short break between each trial, and Zanesco et al. (2020) recorded 16 contiguous 1 min blocks alternating eyes closed and eyes open conditions. Based on these differential findings, it is clear that a thorough description of experimental procedures and a systematic comparison of EEG resting-state activity in different recording conditions will be necessary to provide a coherent view. Unsurprisingly, simply stating that recordings were performed with the eyes closed or the eyes open does not appear to be sufficient.

#### Microstates A and B

We found no age-related differences of map A and B parameters (GEV, mDur and Occ) in either eye condition. Accordingly, Tomescu et al. (2018) reported the mean duration and occurrence of the four canonical microstates (A, B, C and D) and did not find any differences for maps A or B between 20–30-year-old and 31–60-year-old adults, or between 31–60-year-old and 61–87-year-old adults; they did not compare 20–30-year-old and 61–87-year-old adults. In contrast, Zanesco et al. (2020) found higher GEV and mDur, and a lower Occ for maps A and B in 59–77-year-old as compared to 20–35-year-old adults (with both eye conditions combined). They also found higher transition probabilities toward maps A and B in the older group. Zanesco et al. (2020) recorded 16 continuous 1-min blocks in 153 young adults and 74 older adults. They reported important intra-individual variation and suggested that longer recordings of resting EEG should be used to obtain reliable estimates of microstate dynamics when comparing microstates between individuals. However, using test–retest over two consecutive days, Liu et al. (2020) showed high reliability and individual specificity of microstate characteristics (GEV, mean duration, coverage, occurrences and transition probabilities of the 4 canonical maps) with a duration of data collection between only 2 and 5 min. As mentioned above, longer recording durations may lead to fatigue which might impact microstate dynamics (potentially differentially between young and older adults) and might therefore explain discrepancies between studies. Interestingly, it has been reported that vigilance level is negatively associated with occurrence of map A and B and positively associated with duration of map C and D in 39 adults (mean age 43.7 ± 9.8) and 20 young adults (mean age 26.8 ± 7.6) (Krylova et al. 2020). Detailed comparisons of experimental procedures in various populations will be essential to provide a better understanding of these differences.

Map A has been linked to activity in the left temporal lobe and left insula, whereas map B has been linked to activity in the left and right occipital cortex, which contribute respectively to auditory and visual processing (Custo et al. 2017). It has been shown that brain networks involved in sensory processing exhibit increased functional connectivity with age, whereas higher order processing networks exhibit decreased functional connectivity with age (Geerligs et al. 2015; Tomasi and Volkow 2012). As aging seems to differentially impacts distinct functional networks, some authors have suggested that it might reflect age-related functional reorganization or compensatory mechanisms (Reuter-Lorenz and Park 2010). Although we found age-related decreases in microstates associated with higher order processing networks (see below), we did not find significant differences in microstates A and B, associated with sensory processing.

#### Microstate C

We found a lower occurrence of map C, as well as a lower GEV in the eyes open condition, for older adults as compared to young adults. Similarly, Zanesco et al. (2020) found a lower occurrence and a lower GEV of map C for older adults when both eye conditions were combined. In contrast, Tomescu et al. (2018) did not report age-related differences in the occurrence of map C. Because their global ANOVA did not reveal significant differences between the five age groups tested, they did not specifically test possible differences between 20–30-year-old and 61–97-year-old adults, which might have revealed results similar to Zanesco et al. (2020) and the present report.

We did not find age-related differences for mean duration of map C. In contrast, Tomescu et al. (2018) using the four canonical microstates (A, B, C, D) reported a longer mean duration of map C (which might be a combination of maps C and C’ when more maps are computed, Michel and Koenig 2018) in 31–60-year-old adults as compared to 20–30-year-olds, and no difference between 31–60-year-old and 61–97-year-old adults. Similarly, Zanesco et al. (2020) using a data-driven approach and computing five maps (A, B, C, D, E), also reported a longer mean duration of map C in 59–77-year-old adults as compared to 20–35-year-old adults. Although some discrepancies exist between studies considering map C mean duration across ages, both the current study and Zanesco’s study, using a data driven approach, showed a lower occurrence and GEV of map C in older adults as compared to young adults, suggesting a decreased activity in its underlying networks.

Map C, as computed in our study (Michel and Koenig 2018), has been linked to neuronal activity in parietal brain regions, in particular the precuneus (PrC) and the posterior cingulate cortex (PCC), which are core regions of the default mode network (DMN; Custo et al. 2017). Interestingly, Seitzman et al. (2017) showed that microstate C decreases in duration and occurrence during a mental arithmetic task as compared to rest. Milz et al. (2016b) showed that microstate C decreases in duration during object or verbal visualization tasks compared to rest. Brechet et al. (2019) showed that whereas microstate C duration and occurrence decrease during an arithmetic task as compared to rest, it does not change between an autobiographic memory task and rest, supporting the assumption that self-relevant memory retrieval may also predominate during spontaneous mind wandering (Brechet et al. 2019). Furthermore, it has been shown that the PCC and PrC connections within the DMN network exhibit decreased functional connectivity with age (Klaassens et al. 2017). Changes in DMN functional connectivity have been linked to cognitive decline and working memory impairments (Cieri and Esposito 2018). The lower GEV and lower occurrence of map C in older adults might therefore reflect decreased functional connectivity within the PCC/PrC node and contribute to cognitive decline with age. However, we did not find a direct link between age-related changes in resting-state map C characteristics and spatial working memory decline (see Sect. 4.2).

#### Microstate C′

We found a lower GEV and occurrence of map C′ in older adults as compared to young adults. Similarly, Zanesco et al. (2020) found a lower GEV and occurrence of map E, which largely corresponds to our map C′, in 59–77-year-old adults as compared to 20–35-year-old adults. In addition, they found that even though map E occurred less often, its mean duration was longer in older adults than in young adults (see Sects. 4.1.2 and 4.1.3 for similar results and methodological considerations).

Microstate C′ (as defined by Michel and Koenig 2018; see Sect. 3.1) has been linked to neuronal activity in the dorsal anterior cingulate cortex (dACC), the superior and middle frontal gyrus and the insula (reported as microstate 3 in Britz et al. 2010; map F in Custo et al. 2017; and map E in Zanesco et al. 2020). The fronto-insular cortex and the dACC have been linked to the salience network (SN; Seeley et al. 2007), whose purported function is to identify the most relevant internal and extrapersonal stimuli (Seeley et al. 2007), engaging the brain’s attentional, working memory and higher-order control processes, while disengaging irrelevant systems, to modulate reactivity (Menon and Uddin 2010). In line with the decreased activity of map C′ with age revealed in our study, it has been shown that the structures comprising the SN exhibit a smaller grey matter volume and a lower functional connectivity in healthy older adults than in young adults (He et al. 2014).

#### Microstate D

We found no age-related differences in map D in the eyes closed condition. Similarly, Tomescu et al. (2018) showed no differences of occurrence or mean duration for map D between 20–30-year-old and 31–60-year-old adults, or between 31–60-year-olds and 61–87-year-olds. In contrast, we found a lower occurrence of map D in older adults in the eyes open condition. Zanesco et al. (2020) also found a lower occurrence of map D in 59–77-year-old adults as compared to 20–35-year-old adults, with both eye conditions combined. In addition, they found that although map D occurred less often, its mean duration was longer in older adults than in young adults (see Sects. 4.1.2 and 4.1.3 for similar results and methodological considerations).

Map D has been linked to neuronal activity in the right superior and middle frontal gyri, the right inferior parietal lobe and the right insula (Custo et al. 2017) and the attention network (Britz et al. 2010; but see also Seitzman et al. 2017). In addition, it has been shown that microstate D strongly increases in duration and occurrence during an arithmetic task involving working memory and attentional processes (Brechet et al. 2019). Although there are some inconsistencies in the literature, an age-related decrease in functional connectivity within the attention network has generally been observed (for a review see Damoiseaux 2017). Our results are thus consistent with a decreased activation of the attentional and working memory network in older adults at rest with the eyes open, as compared to young adults. Nevertheless, we did not find a direct link between age-related differences in resting-state map D occurrence and spatial working memory performance.

#### Maps Transitions

We found lower transition probabilities towards map C and C′ in older adults as compared to young adults, in both eye conditions, and lower transition probabilities toward map D in older adults in the eyes open condition. However, these data might be dependent on the occurrence of map C, C′ and D, which were also lower in older adults. In the eyes closed condition, Tomescu et al. (2018) found lower transition probabilities from maps C to D, and from maps D to C in 61–87-year-old adults as compared to 31–60-year-old adults (findings that were independent of the occurrence of the four maps studied). Their data, however, did not reveal lower transition probabilities between any other map toward map C in 61–87-year-old adults as compared to 31–60-year-old adults, or between 20–30-year-olds and 31–60-year-olds. They did not test differences between 20–30-year-old and 61–87-year-old adults. Consistent with our findings, Zanesco et al. (2020) found lower transition probabilities towards map C and lower transition probabilities from map C towards map E (corresponding to our map C′) in older adults as compared to young adults with both eye conditions combined. In contrast to our findings, Zanesco et al. (2020) also found higher transition probabilities towards maps A and B.

Several studies described above have shown that resting state functional dynamics differ between age groups. As suggested by others, such differences might reflect the changes from a structured functional organization of different brain networks in young adults to a more random functional organization in older adults (Damoiseaux 2017; Geerligs et al. 2015; He et al. 2014; Klaassens et al. 2017; Tomasi and Volkow 2012). Accordingly, Petti et al. (2016) analyzed the EEG of 71 participants between 20 and 63 years of age and showed that network communication and global strength tends to decrease with age suggesting that the functional organization of brain networks becomes less organized and more random during normal aging (Petti et al. 2016). Knyazev et al. (2015), using graph-theoretical analysis on the EEG data of 76 young (18–35 years) and 70 older (51–80 years) participants, showed a lower connectivity in beta and gamma band networks in older adults. This observation also suggested that brain networks become more random with age (Knyazev et al. 2015). Our data showing lower probabilities of transitions toward maps C, C′ and D in older adults similarly need to be further investigated as we cannot rule out the possibility that this effect is dependent on map occurrence. However, in line with findings of others it suggests that changes in the functional dynamics between the salience detection and attention networks potentially contribute to spatial working memory performance.

### Resting‐State EEG Microstates and Spatial Working Memory Performance

Our PCA and multiple regression analyses did not reveal any link between resting-state microstates and allocentric spatial working memory performance in young or older adults. In contrast, it has been suggested that age-related differences in resting-state brain activity in the frequency domain may reflect cognitive decline (Anderson and Perone 2018), although no individual measures of resting-state theta, alpha, beta or gamma activity have been clearly identified that reliably predict individual working memory performance (Anderson and Perone 2018). Interestingly, data in rodents (Ash et al. 2016; Pereira et al. 2015) and humans (Rondina et al. 2016) have shown that the neurobiological basis of allocentric or egocentric spatial working memory performance may differ between young and older adults. Here, however, we did not find this difference to be reflected in resting-state EEG microstates. Accordingly, Zanesco et al. (2020) did not find reliable correlations between microstate parameters and measures of personality, mood or cognitive functions.

## Conclusions

This exploratory study provides a systematic evaluation of age-related differences in resting-state microstates and allocentric spatial working memory performance between healthy young (20–30 years) and older (65–75 years) adults. In line with most previous findings (Koenig et al. 2002; Tomescu et al. 2018; Zanesco et al. 2020), we found age-related differences in resting-state brain activity dynamics. We also showed that age-related differences in EEG microstates depend on the recording conditions (Seitzman et al. 2017; Zanesco et al. 2020). Importantly, we found consistent age-related differences in maps C and C′ (i.e., lower GEV, Occ and transition probabilities), which have been associated with frontal and parietal functional networks. In turn, these networks have been associated with a wide range of age-sensitive cognitive functions such as working memory and attentional processes. However, using a principal component analysis, we did not find any link between PCA-extracted microstate components and spatial working memory performance in young or older adults.

## Supplementary Information

Below is the link to the electronic supplementary material.

DOCX 310 kb Supplementary Material 1

## References

[CR1] Abdi H, Williams LJ (2010). Principal component analysis WIREs. Comput Stat.

[CR2] Anderson AJ, Perone S (2018). Developmental change in the resting state electroencephalogram: insights into cognition and the brain. Brain Cogn.

[CR3] Andreou C (2014). Resting-state connectivity in the prodromal phase of schizophrenia: insights from EEG microstates. Schizophr Res.

[CR4] Ash JA, Lu H, Taxier LR, Long JM, Yang Y, Stein EA, Rapp PR (2016). Functional connectivity with the retrosplenial cortex predicts cognitive aging in rats. Proc Natl Acad Sci USA.

[CR5] Banta Lavenex P, Colombo F, Ribordy Lambert F, Lavenex P (2014). The human hippocampus beyond the cognitive map: evidence from a densely amnesic patient. Front Hum Neurosci.

[CR6] Baradits M, Bitter I, Czobor P (2020). Multivariate patterns of EEG microstate parameters and their role in the discrimination of patients with schizophrenia from healthy controls. Psychiatry Res.

[CR7] Bizon JL, Foster TC, Alexander GE, Glisky EL (2012). Characterizing cognitive aging of working memory and executive function in animal models. Front Aging Neurosci.

[CR8] Brechet L, Brunet D, Birot G, Gruetter R, Michel CM, Jorge J (2019). Capturing the spatiotemporal dynamics of self-generated, task-initiated thoughts with EEG and fMRI. Neuroimage.

[CR9] Britz J, Van De Ville D, Michel CM (2010). BOLD correlates of EEG topography reveal rapid resting-state network dynamics. Neuroimage.

[CR10] Brunet D, Murray MM, Michel CM (2011). Spatiotemporal analysis of multichannel EEG CARTOOL. Comput Intell Neurosci.

[CR11] Buckner RL, Vincent JL (2007). Unrest at rest: default activity and spontaneous network correlations. Neuroimage.

[CR12] Burgess N (2006). Spatial memory: how egocentric and allocentric combine. Trends Cogn Sci.

[CR13] Campbell KL, Schacter DL (2017). Aging and the resting state: is cognition obsolete?. Lang Cogn Neurosci.

[CR14] Cantero JL, Atienza M, Salas RM (2002). Human alpha oscillations in wakefulness, drowsiness period, and REM sleep: different electroencephalographic phenomena within the alpha band. Neurophysiol Clin.

[CR15] Chu C, Wang X, Cai L, Zhang L, Wang J, Liu C, Zhu X (2019). Spatiotemporal EEG microstate analysis in drug-free patients with Parkinson’s disease. Neuroimage Clin.

[CR16] Cieri F, Esposito R (2018). Neuroaging through the lens of the resting state networks. Biomed Res Int.

[CR17] Corsi PM (1972). Human memory and the medial temporal region of the brain. Diss Abstr Int.

[CR18] Custo A, Van De Ville D, Wells WM, Tomescu MI, Brunet D, Michel CM (2017). Electroencephalographic resting-state networks: source localization of microstates. Brain Connect.

[CR19] D’Croz-Baron DF, Baker M, Michel CM, Karp T (2019). EEG microstates analysis in young adults with autism spectrum disorder during resting-state. Front Hum Neurosci.

[CR20] da Cruz JR (2020). EEG microstates are a candidate endophenotype for schizophrenia. Nat Commun.

[CR21] Damborska A, Piguet C, Aubry JM, Dayer AG, Michel CM, Berchio C (2019). Altered electroencephalographic resting-state large-scale brain network dynamics in euthymic bipolar disorder patients. Front Psychiatry.

[CR22] Damoiseaux JS (2017). Effects of aging on functional and structural brain connectivity. Neuroimage.

[CR23] Davis SW, Stanley ML, Moscovitch M, Cabeza R (2017). Resting-state networks do not determine cognitive function networks: a commentary on Campbell and Schacter (2016). Lang Cogn Neurosci.

[CR24] de Bock R, Mackintosh AJ, Maier F, Borgwardt S, Riecher-Rossler A, Andreou C (2020). EEG microstates as biomarker for psychosis in ultra-high-risk patients. Transl Psychiatry.

[CR25] Drissi NM (2016). Altered brain microstate dynamics in adolescents with narcolepsy. Front Hum Neurosci.

[CR26] Drissi NM (2019). Corrigendum: altered brain microstate dynamics in adolescents with narcolepsy. Front Hum Neurosci.

[CR27] Engle RW, Tuholski SW, Laughlin JE, Conway ARA (1999). Working memory, short-term memory, and general fluid intelligence: a latent-variable approach. J Exp Psychol Gen.

[CR29] Fabiani M (2012). It was the best of times, it was the worst of times: a psychophysiologist’s view of cognitive aging. Psychophysiology.

[CR30] Folstein MF, Folstein SE, McHugh PR (1975). "Mini-mental state”. A practical method for grading the cognitive state of patients for the clinician. J Psychiatr Res.

[CR31] Geerligs L, Renken RJ, Saliasi E, Maurits NM, Lorist MM (2015). A brain-wide study of age-related changes in functional connectivity. Cereb Cortex.

[CR32] Giordano GM (2018). Neurophysiological correlates of Avolition-apathy in schizophrenia: a resting-EEG microstates study. Neuroimage Clin.

[CR33] Gonzalez-Villar AJ, Trinanes Y, Gomez-Perretta C, Carrillo-de-la-Pena MT (2020). Patients with fibromyalgia show increased beta connectivity across distant networks and microstates alterations in resting-state electroencephalogram. Neuroimage.

[CR34] Gschwind M (2016). Fluctuations of spontaneous EEG topographies predict disease state in relapsing-remitting multiple sclerosis. Neuroimage Clin.

[CR35] He X (2014). Abnormal salience network in normal aging and in amnestic mild cognitive impairment and Alzheimer’s disease. Hum Brain Mapp.

[CR36] Ishihara S (1917). Tests for colour-blindness.

[CR37] Jagust W (2013). Vulnerable neural systems and the borderland of brain aging and neurodegeneration. Neuron.

[CR38] Jia H, Yu D (2019). Aberrant intrinsic brain activity in patients with autism spectrum disorder: insights from EEG microstates. Brain Topogr.

[CR39] Khanna A, Pascual-Leone A, Farzan F (2014). Reliability of resting-state microstate features in electroencephalography. PLoS ONE.

[CR40] Khanna A, Pascual-Leone A, Michel CM, Farzan F (2015). Microstates in resting-state EEG: current status and future directions. Neurosci Biobehav Rev.

[CR41] Kikuchi M (2011). EEG microstate analysis in drug-naive patients with panic disorder. PLoS ONE.

[CR42] Kindler J, Hubl D, Strik WK, Dierks T, Koenig T (2011). Resting-state EEG in schizophrenia: auditory verbal hallucinations are related to shortening of specific microstates. Clin Neurophysiol.

[CR43] Klaassens BL, van Gerven JMA, van der Grond J, de Vos F, Moller C, Rombouts S (2017). Diminished posterior precuneus connectivity with the default mode network differentiates normal aging from Alzheimer’s disease. Front Aging Neurosci.

[CR44] Klencklen G, Banta Lavenex P, Brandner C, Lavenex P (2017). Working memory decline in normal aging: is it really worse in space than in color?. Learn Motiv.

[CR45] Klencklen G, Banta Lavenex P, Brandner C, Lavenex P (2017). Working memory decline in normal aging: memory load and representational demands affect performance. Learn Motiv.

[CR46] Knyazev GG, Volf NV, Belousova LV (2015). Age-related differences in electroencephalogram connectivity and network topology. Neurobiol Aging.

[CR47] Koenig T, Lehmann D, Merlo MC, Kochi K, Hell D, Koukkou M (1999). A deviant EEG brain microstate in acute, neuroleptic-naive schizophrenics at rest. Eur Arch Psychiatry Clin Neurosci.

[CR48] Koenig T (2002). Millisecond by millisecond, year by year: normative EEG microstates developmental stages. Neuroimage.

[CR49] Koenig T, Stein M, Grieder M, Kottlow M (2014). A tutorial on data-driven methods for statistically assessing ERP topographies. Brain Topogr.

[CR50] Koenig T, Studer D, Hubl D, Melie L, Strik WK (2005). Brain connectivity at different time-scales measured with EEG. Philos Trans R Soc Lond B.

[CR51] Krylova M (2020). Evidence for modulation of EEG microstate sequence by vigilance level. Neuroimage.

[CR52] Lakens D (2013). Calculating and reporting effect sizes to facilitate cumulative science: a practical primer for t-tests and ANOVAs. Front Psychol.

[CR53] Lehmann D (2005). EEG microstate duration and syntax in acute, medication-naive, first-episode schizophrenia: a multi-center study. Psychiatry Res.

[CR54] Lehmann D, Michel CM (2011). EEG-defined functional microstates as basic building blocks of mental processes. Clin Neurophysiol.

[CR55] Lehmann D, Strik WK, Henggeler B, Koenig T, Koukkou M (1998). Brain electric microstates and momentary conscious mind states as building blocks of spontaneous thinking: I. Visual imagery and abstract thoughts. Int J Psychophysiol.

[CR56] Liu J, Xu J, Zou G, He Y, Zou Q, Gao JH (2020). Reliability and individual specificity of EEG microstate characteristics. Brain Topogr.

[CR57] Menon V, Uddin LQ (2010). Saliency, switching, attention and control: a network model of insula function. Brain Struct Funct.

[CR58] Michel CM, Koenig T (2018). EEG microstates as a tool for studying the temporal dynamics of whole-brain neuronal networks: a review. Neuroimage.

[CR59] Michel CM, Koenig T, Brandeis D, Gianotti L, Wackermann J (2009). Electrical neuroimaging.

[CR60] Milz P, Faber PL, Lehmann D, Koenig T, Kochi K, Pascual-Marqui RD (2016). The functional significance of EEG microstates—associations with modalities of thinking. Neuroimage.

[CR61] Milz P, Pascual-Marqui RD, Lehmann D, Faber PL (2016). Modalities of thinking: state and trait effects on cross-frequency functional independent brain networks. Brain Topogr.

[CR62] Morcom AM, Fletcher PC (2007). Does the brain have a baseline? Why we should be resisting a rest. Neuroimage.

[CR63] Murphy M, Stickgold R, Ongur D (2020). Electroencephalogram microstate abnormalities in early-course psychosis. Biol Psychiatry Cogn Neurosci Neuroimaging.

[CR64] Murphy M (2020). Abnormalities in electroencephalographic microstates are state and trait markers of major depressive disorder. Neuropsychopharmacology.

[CR65] Musaeus CS, Nielsen MS, Hogh P (2019). Microstates as disease and progression markers in patients with mild cognitive impairment. Front Neurosci.

[CR66] Nishida K (2013). EEG microstates associated with salience and frontoparietal networks in frontotemporal dementia, schizophrenia and Alzheimer’s disease. Clin Neurophysiol.

[CR67] Pascual-Marqui RD, Michel CM, Lehmann D (1995). Segmentation of brain electrical activity into microstates: model estimation and validation. IEEE Trans Biomed Eng.

[CR68] Pereira IT, Gallagher M, Rapp PR (2015). Head west or left, east or right: interactions between memory systems in neurocognitive aging. Neurobiol Aging.

[CR69] Petti M, Toppi J, Babiloni F, Cincotti F, Mattia D, Astolfi L (2016). EEG resting-state brain topological reorganization as a function of age. Comput Intell Neurosci.

[CR70] Pipinis E, Melynyte S, Koenig T, Jarutyte L, Linkenkaer-Hansen K, Ruksenas O, Griskova-Bulanova I (2017). Association between resting-state microstates and ratings on the Amsterdam resting-state questionnaire. Brain Topogr.

[CR71] Portnova GV, Ivanova O, Proskurnina EV (2020). Effects of EEG examination and ABA-therapy on resting-state EEG in children with low-functioning autism. AIMS Neurosci.

[CR72] Raven J, Raven JC, Court JH (2003). Manual for Raven’s progressive matrices and vocabulary scales. Section 1: general overview.

[CR73] Reuter-Lorenz PA, Park DC (2010). Human neuroscience and the aging mind: a new look at old problems. J Gerontol B.

[CR74] Ribordy F, Jabes A, Banta Lavenex P, Lavenex P (2013). Development of allocentric spatial memory abilities in children from 18 months to 5 years of age. Cogn Psychol.

[CR75] Rondina R (2016). Age-related changes to oscillatory dynamics in hippocampal and neocortical networks. Neurobiol Learn Mem.

[CR76] Rowe JW, Kahn RL (1987). Human aging: usual and successful. Science.

[CR77] Sakamoto Y, Ishiguro M, Kitagawa G (1986). Akaike information criterion statistics.

[CR78] Santarnecchi E (2017). EEG microstate correlates of fluid intelligence and response to cognitive training. Brain Topogr.

[CR79] Schlegel F, Lehmann D, Faber PL, Milz P, Gianotti LR (2012). EEG microstates during resting represent personality differences. Brain Topogr.

[CR80] Seeley WW (2007). Dissociable intrinsic connectivity networks for salience processing and executive control. J Neurosci.

[CR81] Seitzman BA, Abell M, Bartley SC, Erickson MA, Bolbecker AR, Hetrick WP (2017). Cognitive manipulation of brain electric microstates. Neuroimage.

[CR82] Smailovic U, Koenig T, Laukka EJ, Kalpouzos G, Andersson T, Winblad B, Jelic V (2019). EEG time signature in Alzheimer s disease: functional brain networks falling apart. Neuroimage Clin.

[CR83] Soni S, Muthukrishnan SP, Sood M, Kaur S, Sharma R (2018). Hyperactivation of left inferior parietal lobule and left temporal gyri shortens resting EEG microstate in schizophrenia. Schizophr Res.

[CR84] Spellman T, Rigotti M, Ahmari SE, Fusi S, Gogos JA, Gordon JA (2015). Hippocampal-prefrontal input supports spatial encoding in working memory. Nature.

[CR85] Strelets V (2003). Chronic schizophrenics with positive symptomatology have shortened EEG microstate durations. Clin Neurophysiol.

[CR86] Tomasi D, Volkow ND (2012) Aging and functional brain networks. Mol Psychiatry 17:471, 549–458. 10.1038/mp.2011.81 10.1038/mp.2011.81 PMC3193908 21727896

[CR87] Tomescu MI (2014). Deviant dynamics of EEG resting state pattern in 22q11.2 deletion syndrome adolescents: a vulnerability marker of schizophrenia?. Schizophr Res.

[CR88] Tomescu MI (2018). From swing to cane: sex differences of EEG resting-state temporal patterns during maturation and aging. Dev Cogn Neurosci.

[CR89] Tomescu MI (2015). Schizophrenia patients and 22q11.2 deletion syndrome adolescents at risk express the same deviant patterns of resting state EEG microstates: a candidate endophenotype of schizophrenia. Schizophr Res Cogn.

[CR90] Vellante F (2020). Euthymic bipolar disorder patients and EEG microstates: a neural signature of their abnormal self experience?. J Affect Disord.

[CR91] Wechsler D (1997). Wechsler adult intelligence scale—third edition.

[CR92] Yoshimura M (2019). Hyperactivation of the frontal control network revealed by symptom provocation in obsessive-compulsive disorder using EEG microstate and sLORETA analyses. Neuropsychobiology.

[CR93] Zanesco AP, King BG, Skwara AC, Saron CD (2020). Within and between-person correlates of the temporal dynamics of resting EEG microstates. Neuroimage.

[CR94] Zappasodi F (2017). Prognostic value of EEG microstates in acute stroke. Brain Topogr.

[CR95] Zappasodi F (2019). EEG microstates distinguish between cognitive components of fluid reasoning. Neuroimage.

