# Supplementary material for: Resting‐State EEG Microstates Parallel Age‐Related Differences in Allocentric Spatial Working Memory Performance
Source: Brain Topogr. 2021 Apr 19;34(4):442–60. doi: 10.1007/s10548-021-00835-3 (PMC8195770; doi:10.1007/s10548-021-00835-3)
Supplement: Supplementary file 1 — DOCX 310 kb Supplementary Material 1 [file 10548_2021_835_MOESM1_ESM.docx]

**Supplementary Material 1. Jabès et al.**

**Clustering solutions for maps A, B, C, C’ and D for each group and eye condition:**

**Older, eyes closed**

**
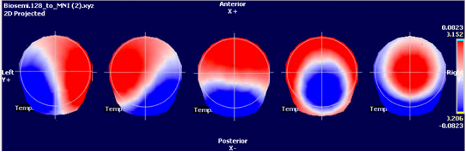
**

**Older, eyes open**

**
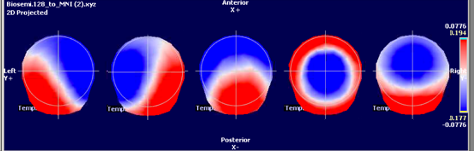
**

**Young, eyes closed**

**
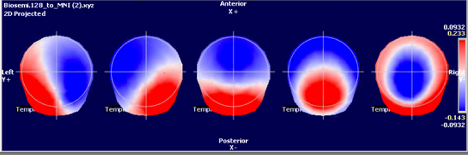
**

**Young, eyes open**

**
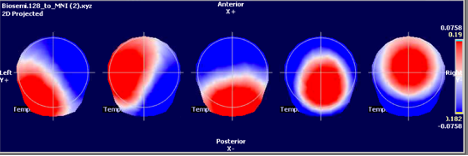
**

**Results of topographic ANOVAs showing no consistent differences between groups or eye conditions:**

**Older: eyes closed vs open**

AA, p = 0.545; BB, p = 0.432; CC, p = 0.838; C’C’, p = 0.176; DD, p = 0.628

**Young: eyes closed vs open**

AA, p = 0.909; BB, p = 0.234; CC, p = 0.350; C’C’, p = 0.173; DD, p = 0.380

**Eyes closed: older vs young**

AA, p = 0.981; BB, p = 0.090; CC, p = 0.026; C’C’, p = 0.154; DD, p = 0.428

**Eyes open: older vs young**

AA, p = 0.532; BB, p = 0.284; CC, p = 0.873; C’C’, p = 0.031; DD, p = 0.491

**Supplementary Material 2. Jabès et al.**

**Descriptive statistics (means and standard deviations) for the five maps: including global explained variance (GEV), mean duration (MDur) and occurrence (occ), for each group (young, older) and eye condition (EC, EO):**

|  | **Young** |  |  |  |  | **Older** |  |  |  |  |
| --- | --- | --- | --- | --- | --- | --- | --- | --- | --- | --- |
|  | **A** | **B** | **C** | **C’** | **D** | **A** | **B** | **C** | **C’** | **D** |
| **GEV EC** | 0.033  (0.019) | 0.075  (0.046) | 0.241  (0.097) | 0.097  (0.057) | 0.069  (0.038) | 0.039  (0.025) | 0.084  (0.036) | 0.183  (0.100) | 0.063  (0.044) | 0.073  (0.039) |
| **GEV EO** | 0.032  (0.017) | 0.050  (0.019) | 0.140  (0.038) | 0.112  (0.043) | 0.083  (0.023) | 0.040  (0.020) | 0.063  (0.025) | 0.107  (0.037) | 0.054  (0.031) | 0.074  (0.023) |
| **MDur EC** | 70.922  (7.797) | 77.176  (9.480) | 99.897  (20.861) | 78.492  (9.447) | 73.799  (7.196) | 72.124  (7.143) | 80.005  (10.227) | 92.026  (17.652) | 76.118  (11.262) | 76.360  (7.404) |
| **MDur EO** | 64.053  (5.948) | 67.354  (3.938) | 79.084  (7.366) | 76.955  (6.040) | 72.444  (4.018) | 67.792  (7.602) | 71.240  (7.842) | 76.097  (8.739) | 70.667  (7.292) | 73.060  (7.578) |
| **Occ EC** | 1.151  (0.504) | 1.630  (0.478) | 2.658  (0.283) | 2.077  (0.602) | 1.799  (0.593) | 1.312  (0.515) | 1.835  (0.453) | 2.386  (0.450) | 1.634  (0.553) | 1.793  (0.571) |
| **Occ EO** | 1.270  (0.501) | 1.555  (0.443) | 2.607  (0.314) | 2.403  (0.407) | 2.201  (0.374) | 1.327  (0.566) | 1.696  (0.584) | 2.234  (0.634) | 1.437  (0.515) | 1.875  (0.468) |

**Descriptive statistics (means and standard deviations) for the spatial working memory task:**

|  | **Young** | **Older** |
| --- | --- | --- |
| **CBE** | 2.225  (0.417) | 1.244  (0.484) |
| **NET** | 6.100  (2.125) | 2.480  (1.828) |

**Supplementary Material 3. Jabès et al.**

**GEV**

**Older adults - eyes open: map C best explained the data**

C vs A, t_(24)_ = 7.790, p < 0.001, d_s_ = 1.558

C vs B, t_(24)_ = 5.177, p < 0.001, d_s_ = 1.035

C vs C’, t_(24)_ = 6.243, p < 0.001, d_s_ = 1.249

C vs D, t_(24)_ = 4.010, p = 0.001, d_s_ = 0.802

**Young adults - eyes open, maps C and C’ best explained the data**

C vs A, t_(19)_ = 11.689, p < 0.001, d_s_ = 2.614

C vs B, t_(19)_ = 9.481, p < 0.001, d_s_ = 2.120

C vs C’, t_(19)_ = 1.949, p = 0.066, d_s_ = 0.436

C vs D, t_(19)_ = 6.028, p < 0.001, d_s_ = 1.348

C’ vs A, t_(19)_ = 7.416, p < 0.001, d_s_ = 1.658

C’ vs B, t_(19)_ = 5.569, p <0.001, d_s_ = 1.245

C’ vs D, t_(19)_ = 2.194, p = 0.041, d_s_ = 0.491

**mDur**

**Older adults - eyes open: map C exhibited the longest mDur**

C vs A, t_(24)_ = 6.320, p < 0.001, d_s_ = 1.264

C vs B, t_(24)_ = 4.024, p < 0.001, d_s_ =0.805

C vs C’, t_(24)_ = 3.939, p = 0.001, d_s_ = 0.788

C vs D, t_(24)_ = 2.638, p = 0.014, d_s_ = 0.528

**Young adults - eyes open: maps C and C’ exhibited the longest mDur**

C vs A, t_(19)_ = 9.521, p < 0.001, d_s_ = 2.129

C vs B, t_(19)_ = 8.408, p < 0.001, d_s_ = 1.880

C vs C’, t_(19)_ = 1.158, p = 0.261, d_s_ = 0.259

C vs D, t_(19)_ = 4.508, p < 0.001, d_s_ = 1.008

C’ vs A, t_(19)_ = 8.842, p < 0.001, d_s_ = 1.977

C’ vs B, t_(19)_ = 7.660, p <0.001, d_s_ = 1.713

C’ vs D, t_(19)_ = 3.651, p = 0.002, d_s_ = 0.816

**Transition probabilities**

**Eyes closed: the probability to transition from any map toward maps C and C’ was overall lower in older adults than in young adults**

AC, t_(43)_ = -2.780 , p = 0.008, d_s_ = 0.834

BC, t_(43)_ = -1.718, p = 0.093, d_s_ = 0.515

C’C, t_(43)_ = -2.672, p = 0.011, d_s_ = 0.802

DC, t_(43)_ = -2.182, p = 0.035, d_s_ = 0.655

AC’, t_(43)_ = -2.238, p = 0.030, d_s_ = 0.671

BC’, t_(43)_ = -2.685, p = 0.010, d_s_ = 0.806

CC’, t_(43)_ = -2.534, p = 0.015, d_s_ = 0.760

DC’, t_(43)_ = -2.826, p = 0.007, d_s_ = 0.848

**Eyes open: the probability to transition from any map toward maps C, C’ and D was overall lower in older adults than in young adult**

AC, t_(43)_ = -2.666, p = 0.011, d_s_ = 0.800

BC, t_(40.870)_ = -2.133, p = 0.039, d_s_ = 0.640

C’C, t_(43)_ = -3.589, p = 0.001, d_s_ = 1.077

DC, t_(43)_ = -2.379, p = 0.022, d_s_ = 0.714

AC’, t_(43)_ = -4.962, p < 0.001, d_s_ = 1.489

BC’, t_(43)_ = -5.184, p < 0.001, d_s_ = 1.555

CC’, t_(43)_ = -6.531, p < 0.001, d_s_ = 1.959

DC’, t_(43)_ = -5.562, p < 0.001, d_s_ = 1.669

AD, t_(43)_ = -2.822, p = 0.007, d_s_ = 0.847

BD, t_(43)_ = -3.085, p = 0.004, d_s_ = 0.926

CD, t_(43)_ = -2.602, p = 0.013, d_s_ = 0.781

C’D, t_(43)_ = -2.772, p = 0.008, d_s_ = 0.832

**The transition probabilities toward maps A, B, C and C' were lower in the eyes open condition than in the eyes closed condition**

BA, t_(44)_ = -2.648, p = 0.011, d_s_ = 0.395

CA, t_(44)_ = -2.736, p = 0.009, d_s_ = 0.408

C’A, t_(44)_ = -3.141, p = 0.003, d_s_ = 0.468

DA, t_(44)_ = -3.504, p = 0.001, d_s_ = 0.522

AB, t_(44)_ = -6.356, p < 0.001, d_s_ = 0.947

CB, t_(44)_ = -6.314, p < 0.001, d_s_ = 0.941

C’B, t_(44)_ = -5.342, p < 0.001, d_s_ = 0.796

DB, t_(44)_ = -4.372, p < 0.001, d_s_ = 0.652

AC, t_(44)_ = -5.712, p < 0.001, d_s_ = 0.851

BC, t_(44)_ = -6.720, p < 0.001, d_s_ = 1.002

C’C, t_(44)_ = -3.789, p < 0.001, d_s_ = 0.565

DC, t_(44)_ = -5.644, p < 0.001, d_s_ = 0.841

AC’, t_(44)_ = -2.136, p = 0.038, d_s_ = 0.318

BC’, t_(44)_ = -2.919, p = 0.006, d_s_ = 0.435

CC’, t_(44)_ = -3.789, p < 0.001, d_s_ = 0.565

DC’, t_(44)_ = -2.966, p = 0.005, d_s_ = 0.442

**The transition probabilities from maps B and C toward map D were lower in the eyes open condition than in the eyes closed condition**

BD, t_(44)_ = -2.378, p = 0.022, d_s_ = 0.354

CD, t_(44)_ = -3.776, p < 0.001, d_s_ = 0.563

**The transitions probabilities from maps A and C’ toward map D did not differ between eye conditions**

AD, t_(44)_ = -1.498, p = 0.141, ds = 0.223

C’D, t_(44)_ = -1.177, p = 0.246, ds = 0.175

**Supplementary Material 4. Jabès et al.**

**Bayesian multiple regression analyses – Bayes factors of the alternative model (association between PCA components and behavior parameters) compared to the null model. A bayes factor < 1 shows that the alternative model is less likely than the null model**

|  | **CBE**  **BF10** | **NET**  **BF10** |
| --- | --- | --- |
| Age group | 431198.515 | 20747.773 |
| Comp. 1 | 0.811 | 2.679 |
| Comp. 2 | 3.610 | 1.474 |
| Comp. 3 | 0.311 | 0.306 |
| Comp. 4 | 0.655 | 0.553 |
| Comp. 5 | 0.393 | 0.388 |

**Bayesian multiple regression analyses – 95% credible interval of the posterior estimates on the PCA components. Evidence for null hypothesis if the interval includes “0”.**

| **CBE Posterior Summaries of Coefficients** | | | | | | | | | | | | | | | |
| --- | --- | --- | --- | --- | --- | --- | --- | --- | --- | --- | --- | --- | --- | --- | --- |
|  | | | | | | | | | | | | **95% Credible Interval** | | | |
| **Coefficient** | | **Mean** | | **SD** | | **P(incl)** | | **P(incl\|data)** | | **BF _inclusion_** | | **Lower** | | **Upper** | |
| Intercept |  | 1.680 |  | 0.070 |  | 1.000 |  | 1.000 |  | 1.000 |  | 1.549 |  | 1.832 |  |
| AGE |  | -0.018 |  | 0.003 |  | 0.500 |  | 1.000 |  | 37453.356 |  | -0.025 |  | -0.012 |  |
| Comp1 |  | 0.002 |  | 0.007 |  | 0.500 |  | 0.210 |  | 0.265 |  | -0.005 |  | 0.030 |  |
| Comp2 |  | -0.004 |  | 0.013 |  | 0.500 |  | 0.234 |  | 0.306 |  | -0.041 |  | 0.017 |  |
| Comp3 |  | -0.002 |  | 0.011 |  | 0.500 |  | 0.198 |  | 0.246 |  | -0.041 |  | 0.014 |  |
| Comp4 |  | -0.008 |  | 0.018 |  | 0.500 |  | 0.295 |  | 0.418 |  | -0.065 |  | 0.004 |  |
| Comp5 |  | -0.004 |  | 0.017 |  | 0.500 |  | 0.214 |  | 0.273 |  | -0.044 |  | 0.030 |  |
|  | | | | | | | | | | | | | | | |

| **NET Posterior Summaries of Coefficients** | | | | | | | | | | | | | | | |
| --- | --- | --- | --- | --- | --- | --- | --- | --- | --- | --- | --- | --- | --- | --- | --- |
|  | | | | | | | | | | | | **95% Credible Interval** | | | |
| **Coefficient** | | **Mean** | | **SD** | | **P(incl)** | | **P(incl\|data)** | | **BF _inclusion_** | | **Lower** | | **Upper** | |
| Intercept |  | 0.409 |  | 0.030 |  | 1.000 |  | 1.000 |  | 1.000 |  | 0.351 |  | 0.468 |  |
| AGE |  | -0.006 |  | 0.001 |  | 0.500 |  | 1.000 |  | 2808.494 |  | -0.009 |  | -0.004 |  |
| Comp1 |  | 0.004 |  | 0.006 |  | 0.500 |  | 0.457 |  | 0.840 |  | -2.263e -4 |  | 0.019 |  |
| Comp2 |  | -0.003 |  | 0.006 |  | 0.500 |  | 0.300 |  | 0.428 |  | -0.021 |  | 0.002 |  |
| Comp3 |  | -8.268e -4 |  | 0.005 |  | 0.500 |  | 0.223 |  | 0.286 |  | -0.015 |  | 0.010 |  |
| Comp4 |  | -0.003 |  | 0.007 |  | 0.500 |  | 0.285 |  | 0.398 |  | -0.020 |  | 0.008 |  |
| Comp5 |  | -0.002 |  | 0.007 |  | 0.500 |  | 0.240 |  | 0.316 |  | -0.023 |  | 0.006 |  |
|  | | | | | | | | | | | | | | | |
